# Predicted choice and acceptability of regimens for tuberculosis preventive treatment among people living with HIV in Uganda – A discrete choice experiment

**DOI:** 10.1371/journal.pgph.0005630

**Published:** 2026-09-10

**Authors:** Hélène E. Aschmann, Allan Musinguzi, Jillian L. Kadota, Catherine Namale, Juliet Kakeeto, Jane Nakimuli, Lydia Akello, Fred Welishe, Anne Nakitende, Christopher Berger, David W. Dowdy, Adithya Cattamanchi, Fred C. Semitala, Andrew D. Kerkhoff

**Affiliations:** 1 Division of Pulmonary and Critical Care Medicine, University of California San Francisco, San Francisco, California, United States of America; 2 Center for Tuberculosis, University of California San Francisco, San Francisco, California, United States of America; 3 Infectious Diseases Research Collaboration, Kampala, Uganda; 4 Uganda Tuberculosis Implementation Research Consortium, Walimu, Kampala, Uganda; 5 Department of Epidemiology, Johns Hopkins Bloomberg School of Public Health, Baltimore, Maryland, United States of America; 6 Division of Pulmonary Diseases and Critical Care Medicine, University of California Irvine, Irvine, California, United States of America; 7 Department of Medicine, Makerere University College of Health Sciences, Kampala, Uganda; 8 Makerere University Joint AIDS Program, Kampala, Uganda; 9 Division of HIV, Infectious Diseases, and Global Medicine, University of California San Francisco, San Francisco, California, United States of America; University of Sydney, AUSTRALIA

## Abstract

Little is known about people living with HIV’s preferences for different tuberculosis preventive treatment (TPT) regimens, or the conditions under which they would accept treatment. Actionable evidence regarding preference for TPT is needed to inform policy and the development of novel TPT regimens. Adults engaged in care at an HIV clinic in Kampala, Uganda, completed a discrete choice experiment survey with nine random choice tasks. In each task, participants first chose between two hypothetical TPT regimens with differing treatment features (number of pills, frequency, duration, adjusted antiretroviral dosage, and side effects). Second, they answered if they would accept the selected treatment, versus taking no treatment. We simulated predicted TPT regimen choice based on hierarchical Bayesian estimation of individual preference weights. Among 400 participants, 394 gave high-quality answers and were included (median age 44, 71.8% female, 91.4% previously received TPT). Across nine tasks, 60.2% (237/394) accepted all selected TPT regimens, 39.3% (155/394) accepted some regimens, and 0.5% (2/394) accepted none. Choice simulations showed that if only 6 months of daily isoniazid (6H) was available, 11.9% of participants were predicted to prefer no TPT. However, offering a 4-pill, fixed-dose combination 3HP regimen in addition to 6H increased the acceptability from 88.1% to 98.8% (predicted choice of 3HP 94.5%, 6H 4.4%, no TPT 1.2%). Choice simulations showed that 10 pills per dose and antiretroviral dosage adjustment had the largest effects on reducing the acceptability of regimens. While adults living with HIV in Uganda demonstrate a high willingness to accept different TPT regimens, offering regimens with preferred features, such as 3HP as a fixed-dose combination, could drive TPT acceptance and uptake from high to nearly universal.

## Introduction

People living with HIV (PLHIV) in high tuberculosis (TB) incidence settings should receive TB preventive treatment (TPT) [1]. However, little is known under which circumstances people would prefer TPT or no TPT, as well as the specific regimens they would accept or reject. In 2024, 56% of PLHIV globally who newly initiated antiretroviral therapy (ART) received TPT [2]. Offering short-course regimens as an alternative to six months of isoniazid (6H) is a key component of more person-centered TB prevention, aligning with the first pillar of the End TB Strategy [3]. The World Health Organization (WHO) recommends several different short-course TPT regimens for PLHIV [1]; however, the availability of these regimens differs by country [4].

In Uganda, national guidelines recommend one of three TPT regimens for adult PLHIV: 6H, three months of weekly isoniazid and rifapentine (3HP), and one month of daily isoniazid and rifapentine (1HP) [5,6]. In practice, only 6H and 3HP are available. While published TPT completion rates among PLHIV in Uganda are typically above 90% [7,8], completion in actual practice is believed to be substantially lower. For example, completion of isoniazid preventive therapy in northeastern Uganda was reported to be 32%, with over half of non-completions due to drug stockouts.[9] Person-centered strategies remain necessary to ensure high coverage among people newly enrolled in HIV care in Uganda [2]. In 2024, of the estimated 99 000 incident TB cases in Uganda, 33% were among PLHIV [2]. Furthermore, the duration of protection provided by TPT and the efficacy of repeat courses of TPT remain uncertain [1,10,11]. Thus, understanding the acceptability of TPT regimens among all PLHIV in Uganda is crucial.

Although a few studies have described the preferences of children and their caregivers [12,13], evidence remains limited on the features and regimens influencing under what conditions PLHIV would accept TPT. Our prior work in Uganda showed that adult PLHIV preferred 3HP over 1HP [14], and highlighted that features beyond duration, including pill burden, dosing frequency, and the need for dosing adjustments to ART significantly influenced regimen choice [15]. Similarly, for children, adolescents, and their caregivers, features like pill size, taste, and packaging have also been shown to be important [13]. However, actionable evidence on expected choices of PLHIV between TPT or no TPT in the context of different regimen options, including those not yet introduced, is needed to guide policy and guideline recommendations.

Therefore, the objectives of this study were to 1) simulate the choice of TPT versus no TPT depending on the availability of different TPT regimens using data from a recent discrete choice experiment (DCE) among adult PLHIV in Uganda and 2) estimate the impact of different regimen features on TPT acceptability. We have previously reported the main effects and heterogeneity of preferences measured in this discrete choice experiment [15], and aim to provide further insights into the impact of regimen characteristics on the acceptability of TPT in this simulation study.

## Methods

### Ethics statement

The Makerere University School of Public Health Research and Ethics Committee (SPH-2022–231), the University of California San Francisco Institutional Review Board (338451) and the Uganda National Council for Science and Technology (HS2261ES) approved the study. All participants provided written informed consent.

### Inclusivity in global research

Additional information regarding the ethical, cultural, and scientific considerations specific to inclusivity in global research is included in the Supporting Information (S1 File).

### Study overview

In this secondary analysis of a discrete choice experiment among adult PLHIV in Uganda, we used choice simulations to predict the acceptability of, and preferences among, existing TPT regimens (Table 1), including those locally available in Uganda, as well as hypothetical future options.

**Table 1:** Simulation parameters of TPT regimens for choice predictions. 1HP: 1 month of daily isoniazid-rifapentine, 3HP: 3 months of weekly isoniazid-rifapentine, 3HR: 3 months of daily isoniazid-rifampin, 4R: 4 months of daily rifampin, 6H: 6 months of daily isoniazid, ART: Antiretroviral therapy, FDC: fixed-dose combination, TPT: Tuberculosis preventive treatment.

TPT	Duration [months]	Frequency	Pills per dose^a^	Adjust ART dosage	Mild side effects [%]^c^	Moderate side effects [%]^c^	Local availability
1HP	1	Daily	4	Yes^b^	57.0	16.8	No^d^
3HP FDC	3	Weekly	4	No	11.5	6.0	Yes
3HP	3	Weekly	10	No	11.5	6.0	Yes
3HR	3	Daily	4	Yes	29.7	2.3	No
4R	4	Daily	1	Yes	20.0	1.7	No
6H	6	Daily	2	No	36.1	8.2	Yes

^a^Pills per dose includes recommended pills of vitamin B6 if applicable.

^b^As definitive studies are lacking, we also performed sensitivity analyses where 1HP was assumed to require no ART dosage adjustment.

^c^Mild side effects were described as transient nausea, dizziness, fatigue, or skin rashes, for about a week, resolving on their own. Moderate or severe side effects were described as bothersome symptoms like tingling feet, allergy, or liver damage, requiring additional visits to the health care facility and which may require stopping medicine or being admitted to the hospital.

^d^1HP is recommended in Ugandan guidelines for people living with HIV but not currently available.

### Setting and participants

We conducted a cross-sectional DCE survey from August 26 to November 23, 2022, as previously described [15]. The study took place at the Mulago Immune Suppression Syndrome (ISS) clinic, in Kampala, Uganda, the largest outpatient HIV clinic in the country. Briefly, individuals were eligible if they were enrolled in HIV care at the clinic, were 18 years or older, had not initiated a TPT regimen in the past year, and were not currently receiving TB treatment. We excluded individuals who did not provide informed consent or were currently in prison.

### Procedures

Consecutive potential participants were approached in the clinic waiting area by an HIV peer counsellor, with additional efforts to recruit persons who had never taken TPT and men. Participants received 20,000 Ugandan Shillings (about 5 USD). The survey was administered by trained interviewers on an electronic tablet. Before starting, participants were provided with general information on latent TB, and the DCE attributes and levels were explained with a structured flipbook, followed by a practice survey task.

### DCE design

The DCE was designed in Sawtooth Lighthouse Studio version 9.13.2 [16], with a list of attributes initially informed by the literature and iteratively refined through pilot testing with 29 patients [15]. The final design included six attributes with 2 or 3 levels each: number of pills (1, 5, or 10 pills), frequency of dosing (weekly, twice per week, or daily), regimen duration (1, 3, or 6 months), adjusted HIV antiretroviral dosage (yes - second dose of ART needed, or no - no adjustment needed), mild side effects (10%, 50%, or 90% risk), and moderate or severe side effects (1%, 10%, or 20% risk) (S1 Fig). Each participant completed nine random choice tasks and two fixed choice tasks: (1) a dominant choice task to assess comprehension of the exercise [17] (S1 Table), and (2) a task approximating 1HP and 3HP (S2 Table). Choice tasks were unlabeled (Treatment “A” and “B”). Participants were randomly allocated to one of 500 randomly generated sets of the nine random choice tasks, using the “balanced overlap” method to allow for analysis of interaction terms.

Participants first selected their preferred TPT regimen (A or B), and were then asked to choose between their preferred regimen and no TPT:

“*If the TB prevention treatment* *you chose was available, would you actually take it? No treatment would mean*:

- Your risk of developing TB is high. About 5 in 100 persons will get TB in the next three years.
- No additional tablets, just continue your usual ART.”

We based our estimate of TB incidence (5 in 100) among PLHIV on ART without TPT in Uganda on incidence rates reported in Uganda [18], a trial investigating early ART initiation without TPT [19], and the observation that TB incidence is about half as high for PLHIV on ART for longer than three years [20]. Similar rates have since been reported based on registry data in Uganda [21]. Participants with prior TPT experience were told: “*Some researchers are evaluating whether it is beneficial to repeat tuberculosis preventive therapy annually or at longer intervals. If it is proven beneficial and you were asked to take the therapy again, we are interested in understanding your preferences around the treatment regimen.*”

As previously reported, we targeted a sample size of 400 participants to allow for robust subgroup analyses [22].

### Statistical analysis

Simulations were performed using Lighthouse Studio version 9.13.2 (Sawtooth Software) [23], and regressions were performed using R version 4.1.2 [24]. Prior to analysis, we conducted data cleaning to ensure the quality of individual-level respondent preference data. Participants with poor response quality – identified through incorrect answers to the dominant fixed choice task or other indications of inattention or incomprehension – were excluded from the analysis, as previously described [15]. We analyzed which features made TPT regimens unacceptable in two ways: by using choice simulations, and by mixed-effects logistic regression to account for individual-level variability [25].

For choice simulations, we used a hierarchical Bayesian model to calculate mean preference weights (i.e., part-worth utilities) for each attribute level. These weights are estimated by assuming that (1) each participant has their own set of preference weights, and (2) these individual preferences are drawn from a broader distribution across the sample, which in turn informs each participant's estimates. Using these preference weights, we applied a Shares of Preference Model using the Sawtooth Choice Simulator tool [26] to predict participants’ choices. The Shares of Preference Model computes the utility for each regimen (adding up participants’ individual part-worth utilities), converts the utilities into choice probabilities, and aggregates the probabilities across all participants to predict which proportion of the study population would choose which regimen. Parameters for existing regimens were defined as shown in Table 1. We simulated 1HP both with and without ART dosage adjustment required, as pharmacokinetic data suggest twice-daily dosing of dolutegravir, though definitive studies are lacking [27]. Likelihoods of side effects were informed by previously reported adverse event rates [28]. We performed choice simulations to: (1) predict preference for TPT over no TPT when only one attribute is changed at a time, using an ideal regimen and 3HP as references; (2) compare preferences for existing regimens; and (3) predict choices for the regimens historically available in Uganda. We selected 3HP as a reference regimen because this is increasingly becoming the standard TPT regimen in Uganda [5]. Additionally, we simulated a scenario where 1HP was introduced alongside 6H and 3HP in the future.

To confirm insights from choice simulations on the impact of individual attributes, we performed regression analyses. TPT regimens were categorized as either “acceptable” (i.e., the selected regimen was preferred over “no treatment”) or “unacceptable” (“no treatment” was preferred over the selected regimen), for each task. If no treatment was preferred over the selected regimen, both presented regimens in the task were classified as “unacceptable”. When a regimen was selected as preferred over the alternative regimen, and as preferred over no treatment, it was classified as “acceptable.” The alternative, less preferred regimen was not included in the regression, as it was unclear whether it would be preferred over no treatment. To minimize sparse data bias, variable categories (e.g., 1 and 5 pills) were grouped so that each combination was classified as acceptable or unacceptable at least 3 times. Side effects were only included in a sensitivity analysis for the same reason. Thus, the mixed effects logistic regression included four fixed effects (duration, frequency, number of pills per dose, and need to adjust ART medications, each with two categories) and a random intercept for individual participants.

## Results

### Participant characteristics

We screened 456 persons, invited 414 to participate, and 401 provided consent. After excluding seven participants due to poor response quality (three failed the dominant task, three were likely inattentive, and one enrolled multiple times with inconsistent answers), we included 394 responses in the analyses. Most participants were female (71.8%), the median age was 44 years, and all were on ART, with a median duration of 10.4 years. Most had previously taken TPT (91.3%), either with 6H (66.7%), 3HP (32.5%) or both (0.8%) (S3 Table).

### Predicted choices for existing regimens

**Fig 1:**
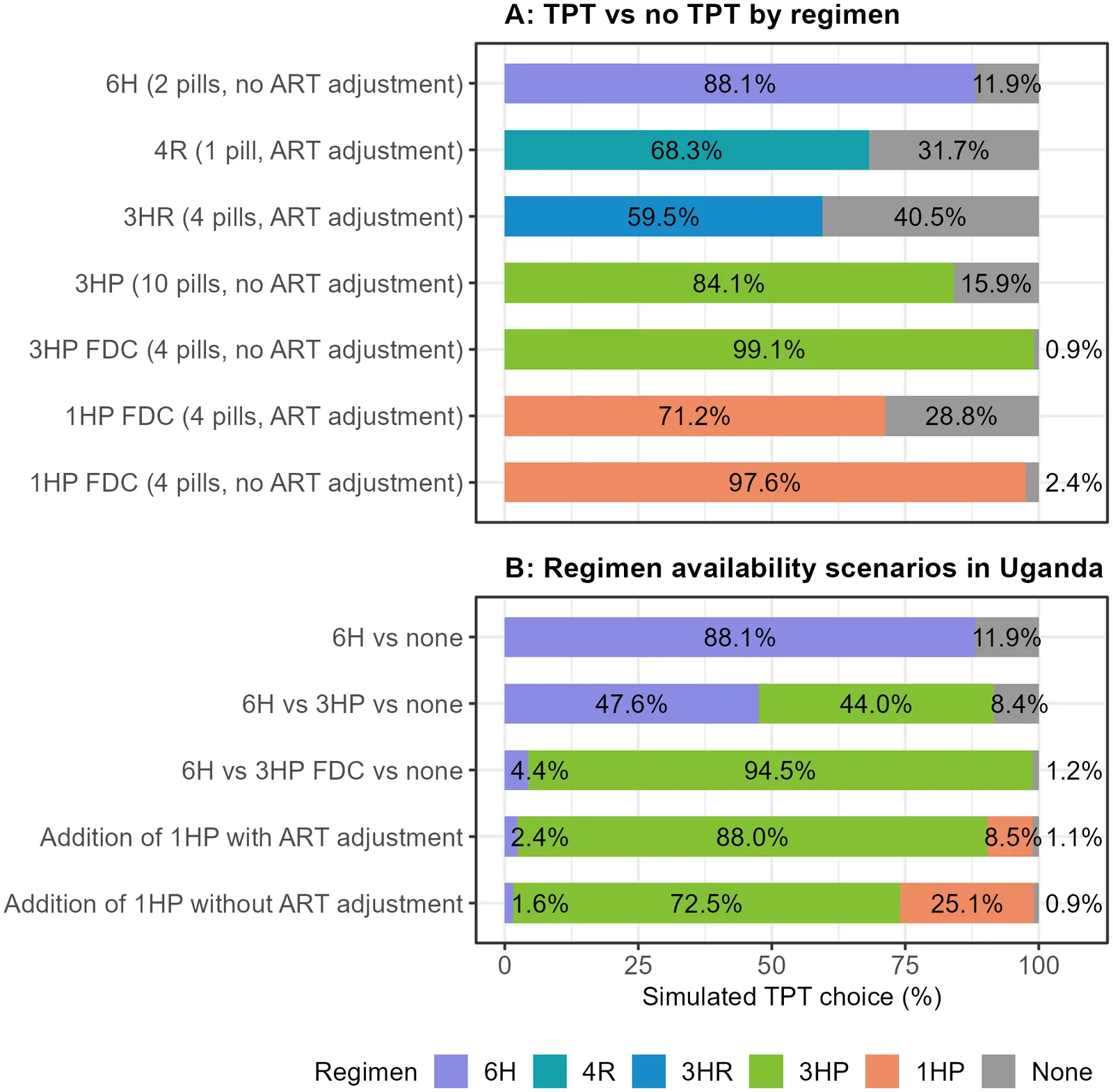
Choice simulations for existing TPT regimens. **A)** Acceptability of single regimens: choice simulation of TPT versus no TPT for all regimens recommended by the World Health Organization. **B)** Historically available regimens in Uganda: Until 2020, only 6H was available. 3HP was first available as a 10- or 11-pill regimen, then as a FDC with 4 pills. This simulation also includes a prediction of choices if 1HP were offered in the future. 1HP: 1 month of daily isoniazid and rifapentine, 3HP: 3 months of weekly isoniazid and rifapentine, 3HR: 3 months of daily isoniazid and rifampin, 4R: 4 months of daily rifampin, 6H: 6 months of daily isoniazid, ART: Antiretroviral therapy, FDC: fixed-dose combination, TPT: tuberculosis preventive therapy.

When comparing the shares of preference for a choice between single existing regimens and no TPT, 3HR, 4R, and 1HP (if requiring ART adjustment) were unacceptable to a large proportion (range, 29–41%) of participants (Fig 1A). The standard 6H regimen was predicted to be preferred over no TPT by 88.1% of participants. The most acceptable regimens were 3HP as a fixed-dose combination (FDC) (4 pills; 99.1% preference over no TPT), and 1HP without ART adjustment (97.6% preference over no TPT).

In simulations of historically available options in Uganda, adding 3HP FDC to 6H reduced no-TPT preference to 1.2%, with 94.5% of participants predicted to choose 3HP (Fig 1B). In two scenarios, offering 1HP in addition to 3HP and 6H, showed little effect on the preference for no TPT. However, 25.1% would prefer 1HP if it required no ART adjustment.

We validated our choice simulations using the fixed choice task representing 3HP and 1HP. The observed preferences (85.0% 3HP, 14.2% 1HP, and 0.7% no TPT) were similar to the predicted probabilities (87.7%, 10.3%, and 2.0%, respectively). Predicted preferences for no TPT did not vary meaningfully by prior TPT experience (S4 Table and S5 Table).

### Impact of TPT features on acceptability

**Fig 2:**
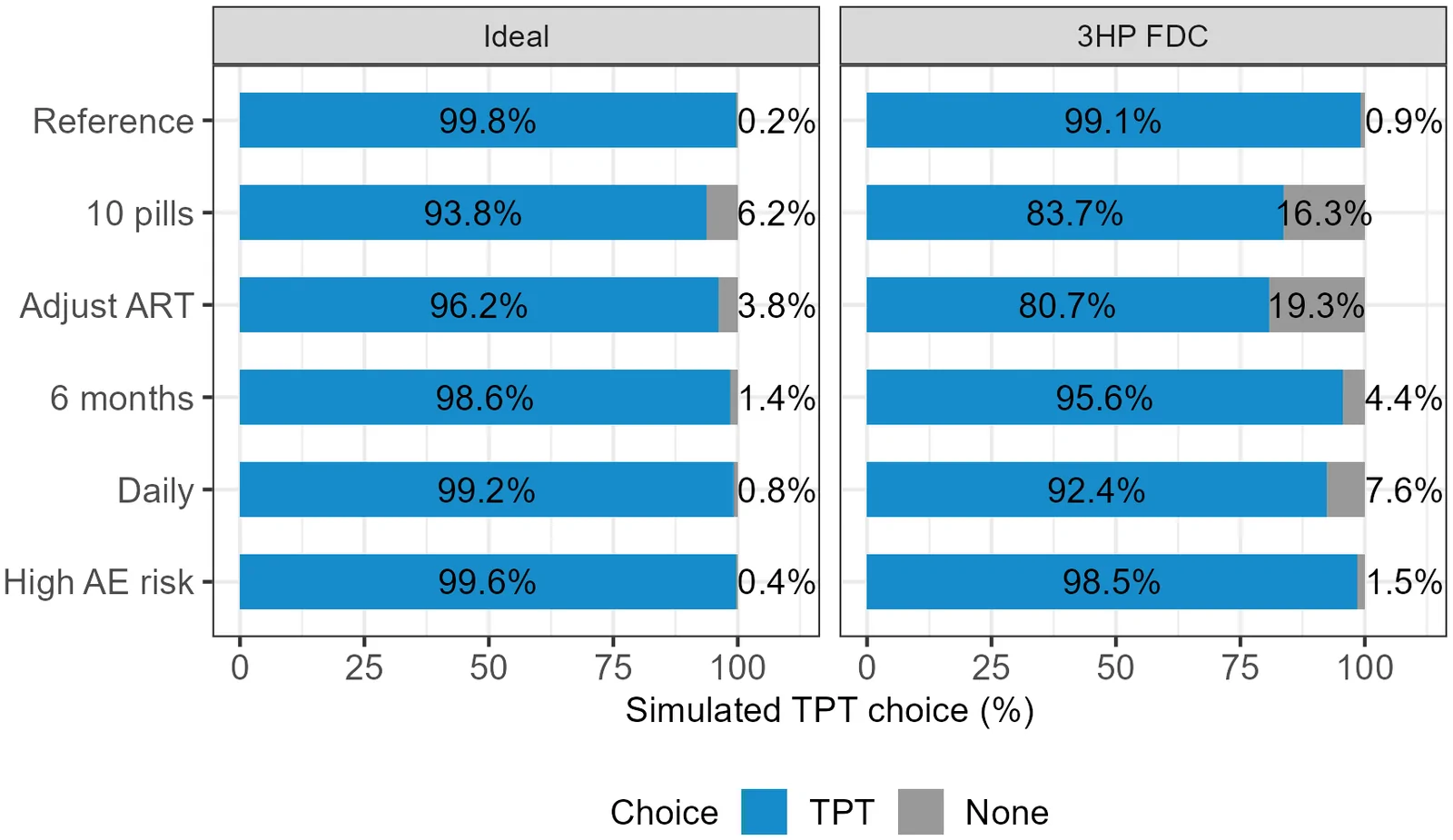
Predicted preference of regimens incorporating least preferred attribute levels in A) an ideal TPT regimen with otherwise optimal features (1 pill per week for 1 month, no ART adjustment, 10% risk of mild side effects, and 1% risk of moderate or severe side effects) and B) in a hypothetical TPT regimen that otherwise mirrors 3HP as FDC (4 pills per week for 3 months, no ART adjustment, 11.5% risk of mild side effects, and 6% risk of moderate or severe side effects). In the last row, “high AE risk”, the frequency of mild side effects was increased to 90% and moderate or severe side effects to 20%. 3HP: 3 months of weekly isoniazid and rifapentine, AE: adverse event, ART: antiretroviral therapy, FDC: fixed-dose combination, TPT: tuberculosis preventive therapy.

In simulations of an ideal TPT regimen (1 pill per dose, no ART adjustment, 1 month duration, weekly frequency, 10% risk of mild side effects, and 1% risk of moderate or severe side effects), 99.8% preferred TPT over no TPT (Fig 2). When modifying the ideal TPT regimen with one undesirable level at a time, 10 pills per dose and ART dosage adjustment had the largest effects: 93.8% would still prefer a 10-pill-per-dose regimen over no TPT, and 96.2% would still prefer TPT if it required ART dosage adjustment. A preference for TPT over no TPT remained above 99% when the regimen required daily dosing or was associated with a high risk of side effects.

In comparison, when 3HP was used as a reference it showed a 99.1% predicted preference over no TPT (Fig 2). If 3HP required ART dosage adjustment, 19.3% would prefer no TPT (18.4% increase in persons not accepting TPT). For a 10-pill 3HP regimen of 3HP, 16.3% would prefer no TPT (15.4% increase in persons not accepting TPT). 3HP was predicted to still be acceptable by more than 90% of participants if it required daily instead of weekly treatment, 6 months instead of 3 months duration, or had a high risk of side effects (90% for mild side effects and 20% for moderate or severe side effects).

Regression analyses utilizing the follow-up dual-none choice question to classify TPT regimens as “acceptable” or “unacceptable” confirmed that 10 pills per dose and ART dosage adjustment were the strongest drivers of acceptability (S6-S8 Table).

## Discussion

In this simulation study using preference data from PLHIV in Uganda, we found that TPT with ART dosage adjustments and high pill burden was often unacceptable to PLHIV, particularly in the context of regimens that contain other less desirable features like longer durations and daily dosing frequency. This suggests that minimizing treatment burden may be more critical for TPT acceptance than duration alone. 3HP as an FDC formulation emerged as the most preferred currently available TPT regimen, with 1HP showing high potential acceptability if ART dose adjustment proves unnecessary like for 3HP. 4R and 3HR, which are less utilized for adults across high burden settings, were predicted not to be acceptable to many participants.

Overall, participants were very willing to take TPT with preferable features, and in particular, 3HP as FDC or 1HP if it does not require ART dosage adjustments [14]. While 1HP or 3HP are now available in more than 28 countries [4], there is limited published evidence about how their introduction has influenced TPT acceptance and uptake across different settings. In Pakistan, two recent studies reported 59% and 72% uptake of 3HP among household contacts in programmatic settings, respectively [29,30], and although uptake was similar for 6H, completion rates were higher for 3HP [30]. Early data from Ethiopia are encouraging, where the introduction of 3HP and its FDC formulation doubled TPT acceptance compared to 6H alone [31].

Several factors likely influence the acceptance and completion of 3HP and other TPT regimens in real-world settings. A qualitative study in Uganda found that fear of contracting TB, awareness of being at risk of TB, and trust in healthcare workers facilitated acceptance and completion of 3HP [32]. Another study in South Africa found that barriers to uptake are reduced after TPT education [33]. Thus, the high acceptability in our study may reflect our educational approach using a pilot-tested education flipbook and our framing of TPT as a tradeoff against a 5% three-year TB risk, as well as familiarity of participants with TPT and specifically 3HP; about 30% of participants had previously taken 3HP, which was introduced at the clinic approximately two years before the present study. A second qualitative study in our setting confirmed that shorter treatment duration and once-weekly dosing facilitated 3HP acceptance, while the high pill burden prior to FDC rollout was a barrier to completing treatment [34]. In contrast to our finding that the risk of adverse events did not impact acceptability, this study also identified perceived 3HP safety as a facilitator for accepting 3HP [34]. This may suggest that recommendations and shared positive experiences from peers and healthcare workers carry greater weight than being presented with varying risks of mild and moderate or severe side effects, which may be difficult for individuals to meaningfully interpret when deciding to start TPT. Similarly, we previously reported that our presentation of side effects did not meaningfully affect the preferences of most participants in our DCE [15], possibly reflecting difficulty of interpreting probability-based risk information, despite our efforts to communicate risk with icon arrays. Unlike in our hypothetical choice, qualitative insights indicate that experiencing side effects can lead to TPT discontinuation, particularly when symptoms interfere with daily activities and income generation [34].

Person-centered preventive care is particularly important to promote uptake among persons who are likely to refuse offered regimens. Prior TPT experience could in principle influence preferences in either direction, and our largely TPT-experienced sample may limit generalizability to TPT-naïve populations; however, we observed no meaningful differences in predicted choices across participants with prior 6H, prior 3HP, or no prior TPT experience. We previously found that participants with fewer years of ART experience and those taking other medications more strongly preferred regimens that did not require ART dosage adjustment [15]. Despite defining ART dosage adjustment as one additional pill of ART per day, its impact on the acceptability of TPT was much larger than for one additional pill per dose of TPT. Future research should explore and examine if alternative ways of explaining or framing ART dosage changes could mitigate some of the negative impacts on TPT acceptability [35].

While offering a choice between 3HP and 1HP did not meaningfully reduce the proportion of participants declining all TPT options, this may reflect our highly treatment-experienced study population, social desirability bias, or other population-specific phenomena. More importantly, offering a choice would improve person-centeredness of TB prevention and likely almost entirely displace the 6H regimen. Notably, if offered 3HP as FDC formulation or 6H (both recommended by Ugandan guidelines), about 84% of individuals would switch from 6H to 3HP. In addition, our findings showed that 25% of those who would accept 3HP would prefer 1HP (with no ART dosage adjustment) if available, demonstrating that providing TPT regimen choices better aligns with individuals’ preferences. These findings have two important consequences from a public health perspective: offering a choice of short-course regimens may increase cost but also increase TPT coverage more strongly than our simulations suggest. Although 3HP is more expensive that 6H, cost-effectiveness analyses suggest that, at current 3HP pricing, 3HP is likely to be cost-effective relative to 6H due to higher completion [36]. Structured choice of 3HP and 1HP or other TPT alternatives that could become available in the future could have a greater impact on acceptability in more TPT-naïve populations and in other settings. Recent findings from a large HIV prevention study in rural Uganda and Kenya demonstrate the potential of such approaches, where offering dynamic choice among post-exposure prophylaxis, oral, and long-acting injectable pre-exposure prophylaxis increased prevention coverage time from 13% to 70% and eliminated incident HIV cases [37]. Similar approaches for TPT have yet to be evaluated, but they could increase population-level coverage by reaching people who might not otherwise accept available regimens [38]. Offering regimen choices may come with challenges for TB and HIV programs regarding implementation, as more counseling and tailored support to complete each regimen may be needed. For example, in the 3HP Options Trial, a decision aid was used to support participants in choosing between directly observed and self-administered therapy to complete 3HP [34,39].

Key strengths of our study include that our choice simulations provide actionable evidence for and against the scale-up of different TPT regimens, the DCE methodology provides a robust approach to measuring preferences, and including an opt-out option after regimen selection enabled us to identify scenarios where even a “preferred” regimen was ultimately unacceptable – an insight that would be missed in forced-choice designs [40–42]. Our study has some limitations. First, we could not validate participants’ hypothetical choices against actual treatment decisions. However, strong alignment between simulated predictions and a fixed choice task comparing 3HP versus 1HP provides some reassurance of response reliability and the predictive properties of the model [43]. Second, despite special recruitment efforts to include TPT naïve persons, 91% of participants had prior TPT. We may have overestimated the acceptability of TPT (as most participants had prior TPT) or underestimated it (as we oversampled persons with no prior TPT). However, the main target audience for new TPT regimens may be people who would not accept current TPT options. Third, our results may not be generalizable to rural areas, newly diagnosed PLHIV, and PLHIV who are not actively engaged in HIV care. Additional research to explore how preferences may vary across such populations would be valuable as access to TPT is expanded in the future. Future studies should also assess additional features that may be relevant to TPT decision-making beyond regimen choice.

## Conclusion

In conclusion, our choice simulations inform which existing TPT regimens should be prioritized to promote uptake and patient-centeredness among PLHIV in Uganda. 3HP as FDC, which is increasingly the standard regimen in Uganda and other high-burden countries, was highly preferred by participants, and its continued rollout should be prioritized. Although offering 1HP alongside 3HP as FDC is not expected to substantially improve overall TPT uptake in our study population, it would enhance patient-centered preventive care by providing an option for those who prefer shorter duration over less frequent dosing, particularly if ART dosage adjustments prove unnecessary. Future research should explore the feasibility of offering and delivering regimen choices, especially as new TPT options with preferred features become available.

## Supporting information

S1 File Checklist: Inclusivity in global research. (DOCX)

S1 Table Levels for dominant choice task. (DOCX)

S2 Table Levels for fixed choice task representing 1HP versus 3HP. (DOCX)

S3 Table Participant characteristics at baseline, based on medical records and self-report. (DOCX)

S4 Table Simulated choice between 6H, 3HP, and no TPT by prior TPT experience. Participants with prior TPT experience were asked to imagine being offered repeat TPT, if it was proven effective.
(DOCX)

S5 Table Simulated choice between 6H and no TPT by prior TPT experience. Participants with prior TPT experience were asked to imagine being offered repeat TPT, if it was proven effective. Thus, the interpretation is that 12.6% of participants with prior experience of 6H would not accept 6H if it was offered as a repeat regimen.
(DOCX)

S6 Table Regimen features associated with unacceptable vs acceptable TPT regimens. The analysis was conducted using mixed effects logistic regression.
(DOCX)

S7 Table Sensitivity analysis for regimen features associated with unacceptable vs acceptable TPT regimens. This sensitivity analysis includes side effects, which were not included in the main analysis to address sparse data bias. The analysis was conducted using mixed effects logistic regression.
(DOCX)

S8 Table Sensitivity analysis for regimen features associated with unacceptable vs acceptable TPT regimens. This sensitivity analysis distinguishes all levels for duration, frequency, and number of pills per dose. Estimates for 10 pills per dose and ART dose adjustment are not robust and have wide confidence intervals. This is due to few observations of some combinations as unacceptable regimen, such as 1 weekly pill for 1 month. Estimates should therefore be interpreted with caution. The main analysis combines some levels to allow for robust estimates.
(DOCX)

S1 Fig Attributes and levels in the discrete choice experiment describing different tuberculosis preventive treatment regimens. This figure shows the final selection of attributes (column 1) and how levels were depicted to participants (columns 2–4). ART: antiretroviral therapy, HIV: human immunodeficiency virusReproduced from Aschmann, Hélène E., et al. “Preferences of people living with HIV for features of tuberculosis preventive treatment regimens in Uganda: a discrete choice experiment.” Journal of the International AIDS Society 27.12 (2024): e26390.
(DOCX)
