# Supplementary material for: Predicted choice and acceptability of regimens for tuberculosis preventive treatment among people living with HIV in Uganda – A discrete choice experiment
Source: PLOS Glob Public Health. 2026 Sep 10;6(9):e0005630. doi: 10.1371/journal.pgph.0005630 (PMC13561322; doi:10.1371/journal.pgph.0005630)
Supplement: Supplementary file 1 [file pgph.0005630.s001.docx]

Inclusivity in global research

**Ethical considerations, permits and authorship**

*This section is applicable to all research types.*

Provide details as to who granted permissions and/or consent for the study to take place in the Methods section of your manuscript. This should include the names of **all** ethics boards, governmental organizations, community leaders or other bodies that provided approval for the study. If individuals provided approval refer to these people by their role or title but do not list their name(s).

Reported on page number: 4, first section in the methods section

If there were any deviations from the study protocol after approval was obtained please provide details of these changes in the Methods section of your manuscript.
Did this study involve local collaborators that are residents of the country where the research was conducted or members of the community studied? If you do not have any authors from said communities, please provide an explanation for this below.

Yes, all local collaborators (AM, FCS, CN, JK, JN, LA, FW, and AN) are included as authors.
This research reflects a collaboration between AM and HEA and was embedded as a sub-study in the 3HP Options trial. AM (in Uganda), the study coordinator of the 3HP Options trial, conducted this study as his master's thesis in public health.

No deviations from the study protocol. Necessary changes were pre-approved by all committees before implementation.

Everyone listed as an author should meet PLOS’ criteria for authorship and all individuals who meet these criteria should be included in the author byline, rather than the acknowledgements. For further information please see the journal’s Authorship Policy.

**Human subjects research (e.g. health research, medical research, cross-cultural psychology)**

Did you obtain written informed consent from a representative of the local community or region before the research took place? How did you establish who speaks for the community? Details of written informed consent obtained from study participants should be reported separately in the Methods section of your manuscript.

The Makerere University School of Public Health Research and Ethics Committee (SPH-2022-231) and the Uganda National Council for Science and Technology (HS2261ES) approved the study (as well as the University of California San Francisco Institutional Review Board, 338451). All participants provided written informed consent.

How did members of the local community provide input on the aims of the research investigation, its methodology, and its anticipated outcome(s)?

No lay members of the community provided input. However, the local research team was closely involved.
HEA, JLK (both in USA) and AM met weekly to discuss the design and the progress of the study. The survey had two parts. AM led the design, analysis, and publication of the first part, a direct comparison between 1HP and 3HP (<https://doi.org/10.5588/ijtldopen.23.0283>). HEA led the design, analysis, and publication of the second part, a discrete choice experiment. FCS, the local PI in Uganda, provided supervision. The Ugandan study team, CN, JK, JN, LA, FW, and AN, provided feedback on the study design during the pilot study and discussion of the findings. Based on their feedback, we dropped an attribute, reduced the number of tasks, and shortened the survey.

When engaging with the local community, how did you ensure that the informed consent documents and other materials could be understood by local stakeholders?

All participants provided written informed consent. The study team was trained in Good Clinical Practice and ensured confidentiality and privacy while conducting the surveys. Study staff was fluent in Luganda and English and the informed consent and survey was offered in both Luganda and English.

Will the findings of the research be made available in an understandable format to stakeholders in the community where the study was conducted (e.g. via a presentation, summary report, copies of publications, etc.)? Please provide details of how this will be achieved.

AM presented these study results at two local conferences (the Joint Annual Scientific Health Conference in Entebbe, Uganda in September 2023 and the Ministry of Health, National TB and Leprosy Research and Innovation Symposium in Kampala, Uganda, in March 2024). AM and FCS also presented these results to the Uganda National TB and Leprosy Program (NTLP) research forum in July 2023.
