# Supplementary material for: Predicted choice and acceptability of regimens for tuberculosis preventive treatment among people living with HIV in Uganda – A discrete choice experiment
Source: PLOS Glob Public Health. 2026 Sep 10;6(9):e0005630. doi: 10.1371/journal.pgph.0005630 (PMC13561322; doi:10.1371/journal.pgph.0005630)
Supplement: Supplementary file 2 [file pgph.0005630.s002.docx]

**Table S1**: Levels for dominant choice task

|  | Treatment A | Treatment B |
| --- | --- | --- |
| Duration | 6 months | 1 month |
| Frequency | Daily | Daily |
| Number of pills | 10 | 1 |
| Adjust ART dosage | No | No |
| Mild side effects | 90% | 10% |
| Moderate side effects | 20% | 1% |

ART: antiretroviral therapy
