# Supplementary material for: Predicted choice and acceptability of regimens for tuberculosis preventive treatment among people living with HIV in Uganda – A discrete choice experiment
Source: PLOS Glob Public Health. 2026 Sep 10;6(9):e0005630. doi: 10.1371/journal.pgph.0005630 (PMC13561322; doi:10.1371/journal.pgph.0005630)
Supplement: Supplementary file 3 [file pgph.0005630.s003.docx]

**Table S2**: Levels for fixed choice task representing 1HP versus 3HP

|  | Treatment A – 1HP | Treatment B – 3HP |
| --- | --- | --- |
| Duration | 1 month | 3 months |
| Frequency | Daily | Weekly |
| Number of pills | 5 | 5 |
| Adjust ART dosage | Yes | No |
| Mild side effects | 50% | 50% |
| Moderate side effects | 20% | 10% |

1HP: 1 month of daily isoniazid and rifapentine, 3HP: 3 months of weekly isoniazid and rifapentine, ART: antiretroviral therapy
