# Supplementary material for: Predicted choice and acceptability of regimens for tuberculosis preventive treatment among people living with HIV in Uganda – A discrete choice experiment
Source: PLOS Glob Public Health. 2026 Sep 10;6(9):e0005630. doi: 10.1371/journal.pgph.0005630 (PMC13561322; doi:10.1371/journal.pgph.0005630)
Supplement: Supplementary file 4 [file pgph.0005630.s004.docx]

**Table S3**: Participant characteristics at baseline, based on medical records and self-report

| Participants (N = 394) | N  median | (%)  (IQR) |
| --- | --- | --- |
| Female sex | 283 | (71.8%) |
| Age | 44 | (IQR: 38, 51) |
| BMI | 26.2 | (IQR: 22.5, 30.1) |
| Education |  |  |
| None | 88 | (22.3%) |
| Primary | 148 | (37.6%) |
| Secondary | 115 | (29.2%) |
| Tertiary or higher | 43 | (10.9%) |
| Work status |  |  |
| Employed | 75 | (19.0%) |
| Self-employed | 240 | (60.9%) |
| Unemployed | 43 | (10.9%) |
| Not working | 22 | (5.6%) |
| Other | 14 | (3.6%) |
| Multidimensional poverty index^1^ | |  |
| Severely poor | 11 | (2.8%) |
| Poor | 59 | (15.0%) |
| Vulnerable | 110 | (27.9%) |
| Not vulnerable | 214 | (54.3%) |
| Prior TPT |  |  |
| None | 34 | (8.6%) |
| Prior 6H | 240 | (60.9%) |
| Prior 3HP | 117 | (29.7%) |
| Both prior 6H and 3HP | 3 | (0.8%) |
| TPT completion (N=360)^2^ |  |  |
| TPT completed | 349 | (96.9%) |
| No, I discontinued LTBI treatment | 10 | (2.5%) |
| Do not know / do not want to answer | 1 | (0.3%) |
| Experienced side effects from TPT (N=360)^2^ | | |
| Yes, and I had to see my doctor about it. | 28 | (7.8%) |
| Yes, but only mild ones and I did not see my doctor about it. | 57 | (15.8%) |
| No, I was fine. | 275 | (76.4%) |
| History of active tuberculosis | 71 | (18.0%) |
| Current antiretroviral therapy |  |  |
| Dolutegravir-based | 372 | (94.4%) |
| Efavirenz-based | 14 | (3.6%) |
| Other | 8 | (2.0%) |
| Time on antiretroviral therapy (years) | 10.4 | (IQR: 7.2,14.1) |
| Viral load |  |  |
| Suppressed | 388 | (98.5%) |
| Unsuppressed (≥1000 copies) | 3 | (0.8%) |
| Not yet done, recent HIV diagnosis | 1 | (0.3%) |
| Missing | 2 | (0.5%) |
| Taking other medications^3^ | 145 | (36.8%) |
| Herbal medicine use |  |  |
| Within last month | 90 | (22.8%) |
| Within last year | 96 | (24.4%) |
| Longer than a year ago | 124 | (31.5%) |
| Never | 84 | (21.3%) |
| Hormonal contraceptives among women (N=283) | 52 | (18.4%) |

1. The multidimensional poverty index captures deprivations in health, education, and living standards.
2. Self-reported completion or side effects among those who reported prior TPT.
3. Currently taking other medications not including HIV medication or contraceptives.

3HP: 3 months of isoniazid and rifapentine, 6H: 6 months of isoniazid, BMI: body mass index, HIV: human immunodeficiency virus, IQR: interquartile range, TPT: tuberculosis preventive therapy.
