# Supplementary material for: Predicted choice and acceptability of regimens for tuberculosis preventive treatment among people living with HIV in Uganda – A discrete choice experiment
Source: PLOS Glob Public Health. 2026 Sep 10;6(9):e0005630. doi: 10.1371/journal.pgph.0005630 (PMC13561322; doi:10.1371/journal.pgph.0005630)
Supplement: Supplementary file 5 [file pgph.0005630.s005.docx]

**Table S4**: Simulated choice between 6H, 3HP, and no TPT by prior TPT experience. Participants with prior TPT experience were asked to imagine being offered repeat TPT, if it was proven effective.

|  | Predicted choice 3HP | Predicted choice 6H | Predicted choice None |
| --- | --- | --- | --- |
| All participants | 94.5% | 4.4% | 1.2% |
| No TPT experience | 91.9% | 4.7% | 3.3% |
| 6H experience | 93.6% | 5.1% | 1.3% |
| 3HP experience | 96.3% | 3.6% | 0.2% |

3HP: 3 months of weekly isoniazid and rifapentine, 6H: 6 months of daily isoniazid, ART: Antiretroviral therapy, TPT: tuberculosis preventive therapy
