# Supplementary material for: Predicted choice and acceptability of regimens for tuberculosis preventive treatment among people living with HIV in Uganda – A discrete choice experiment
Source: PLOS Glob Public Health. 2026 Sep 10;6(9):e0005630. doi: 10.1371/journal.pgph.0005630 (PMC13561322; doi:10.1371/journal.pgph.0005630)
Supplement: Supplementary file 6 [file pgph.0005630.s006.docx]

**Table S5**: Simulated choice between 6H and no TPT by prior TPT experience. Participants with prior TPT experience were asked to imagine being offered repeat TPT, if it was proven effective. Thus, the interpretation is that 12.6% of participants with prior experience of 6H would not accept 6H if it was offered as a repeat regimen.

|  | Predicted choice 6H | Predicted choice None |
| --- | --- | --- |
| All participants | 88.1% | 11.9% |
| No TPT experience | 87.2% | 12.8% |
| 6H experience | 87.4% | 12.6% |
| 3HP experience | 90.1% | 9.9% |

3HP: 3 months of weekly isoniazid and rifapentine, 6H: 6 months of daily isoniazid, ART: Antiretroviral therapy, TPT: tuberculosis preventive therapy
