# Supplementary material for: Predicted choice and acceptability of regimens for tuberculosis preventive treatment among people living with HIV in Uganda – A discrete choice experiment
Source: PLOS Glob Public Health. 2026 Sep 10;6(9):e0005630. doi: 10.1371/journal.pgph.0005630 (PMC13561322; doi:10.1371/journal.pgph.0005630)
Supplement: Supplementary file 7 [file pgph.0005630.s007.docx]

## Regression on acceptability of TPT by attribute levels

TPT regimens were substantially more likely to be considered unacceptable when they required 10 pills per dose compared to 1 or 5 pills (aOR 24.5 [95% CI 16.6 – 36.3]), ART dosage adjustment (aOR 27.4 [95% CI 18.5 – 40.7]), 3 or 6 months duration compared to 1 month (aOR 4.9 [95% CI 3.5 – 6.8]), and more frequent than weekly dosing (aOR 3.4 [95% CI 2.5 – 4.6]) (**Table S6**). In a sensitivity analysis, mild and moderate or severe side effects did not meaningfully influence the acceptability of a regimen and the results for other regimen features remained robust (**Table S7**). In a sensitivity analysis that distinguished all levels for duration, frequency, and number of pills, even regimens with 3-month compared to 1-month durations (aOR 4.0 [95% CI 2.7 – 5.9]), and 5 pills per dose compared to 1 pill per dose (aOR 4.7 [95% CI 3.1 – 7.1]) were more likely to be unacceptable; estimates for 10 vs 1 pill and ART dosage adjustment were unstable due to sparse observations of unacceptable regimens for some combinations (**Table S8**).

**Table S6**: Regimen features associated with unacceptable vs acceptable TPT regimens. The analysis was conducted using mixed effects logistic regression.

|  | aOR | [95% CI] | logOR | SE |
| --- | --- | --- | --- | --- |
| Intercept | - | - | -9.89 | 0.81 |
| Duration |  |  |  |  |
| 3 or 6 months vs 1 month | 4.9 | [3.5, 6.8] | 1.58 | 0.17 |
| Dosing frequency |  |  |  |  |
| More than weekly vs weekly | 3.4 | [2.5, 4.6] | 1.22 | 0.16 |
| Number of pills per dose |  |  |  |  |
| 10 vs 1 or 5 | 24.5 | [16.6, 36.3] | 3.20 | 0.20 |
| ART dosage adjustment vs none | 27.4 | [18.5, 40.7] | 3.31 | 0.20 |
|  |  |  |  | **SD** |
| Random effects (participants) |  |  |  | 4.57 |

ART: antiretroviral therapy, aOR: adjusted odds ratio, logOR: log odds ratio or beta coefficients, SD: standard deviation, SE: standard error
