# Supplementary material for: Predicted choice and acceptability of regimens for tuberculosis preventive treatment among people living with HIV in Uganda – A discrete choice experiment
Source: PLOS Glob Public Health. 2026 Sep 10;6(9):e0005630. doi: 10.1371/journal.pgph.0005630 (PMC13561322; doi:10.1371/journal.pgph.0005630)
Supplement: Supplementary file 9 [file pgph.0005630.s009.docx]

## Regression on acceptability of TPT by attribute levels

TPT regimens were substantially more likely to be considered unacceptable when they required 10 pills per dose compared to 1 or 5 pills (aOR 24.5 [95% CI 16.6 – 36.3]), ART dosage adjustment (aOR 27.4 [95% CI 18.5 – 40.7]), 3 or 6 months duration compared to 1 month (aOR 4.9 [95% CI 3.5 – 6.8]), and more frequent than weekly dosing (aOR 3.4 [95% CI 2.5 – 4.6]) (**Table S6**). In a sensitivity analysis, mild and moderate or severe side effects did not meaningfully influence the acceptability of a regimen and the results for other regimen features remained robust (**Table S7**). In a sensitivity analysis that distinguished all levels for duration, frequency, and number of pills, even regimens with 3-month compared to 1-month durations (aOR 4.0 [95% CI 2.7 – 5.9]), and 5 pills per dose compared to 1 pill per dose (aOR 4.7 [95% CI 3.1 – 7.1]) were more likely to be unacceptable; estimates for 10 vs 1 pill and ART dosage adjustment were unstable due to sparse observations of unacceptable regimens for some combinations (**Table S8**).

**Table S8**: Sensitivity analysis for regimen features associated with unacceptable vs acceptable TPT regimens. This sensitivity analysis distinguishes all levels for duration, frequency, and number of pills per dose. Estimates for 10 pills per dose and ART dose adjustment are not robust and have wide confidence intervals. This is due to few observations of some combinations as unacceptable regimen, such as 1 weekly pill for 1 month. Estimates should therefore be interpreted with caution. The main analysis combines some levels to allow for robust estimates.

|  | aOR | [95% CI] |
| --- | --- | --- |
|  | Mixed effects | |
| Duration |  |  |
| 3 vs 1 month | 4.0 | [2.7, 5.9] |
| 6 vs 1 month | 11.6 | [7.4, 18.3] |
| Dosing frequency |  |  |
| Twice per week vs weekly | 1.8 | [1.2, 2.6] |
| Daily vs weekly | 10.5 | [6.8, 16.3] |
| Number of pills per dose |  |  |
| 5 vs 1 | 4.7 | [3.1, 7.1] |
| 10 vs 1 | 88.7 | [50.1, 157.0] |
| ART dosage adjustment vs none | 57.4 | [34.5, 95.5] |

ART: antiretroviral therapy, aOR: adjusted odds ratio
