# Supplementary material for: Predicted choice and acceptability of regimens for tuberculosis preventive treatment among people living with HIV in Uganda – A discrete choice experiment
Source: PLOS Glob Public Health. 2026 Sep 10;6(9):e0005630. doi: 10.1371/journal.pgph.0005630 (PMC13561322; doi:10.1371/journal.pgph.0005630)
Supplement: Supplementary file 10 [file pgph.0005630.s010.docx]

**Figure S1**: Attributes and levels in the discrete choice experiment describing different tuberculosis preventive treatment regimens. This figure shows the final selection of attributes (column 1) and how levels were depicted to participants (columns 2-4).
ART: antiretroviral therapy, HIV: human immunodeficiency virus

Reproduced from Aschmann, Hélène E., et al. "Preferences of people living with HIV for features of tuberculosis preventive treatment regimens in Uganda: a discrete choice experiment." Journal of the International AIDS Society 27.12 (2024): e26390.
