# Supplementary material for: Predicted choice and acceptability of regimens for tuberculosis preventive treatment among people living with HIV in Uganda – A discrete choice experiment
Source: PLOS Glob Public Health. 2026 Sep 10;6(9):e0005630. doi: 10.1371/journal.pgph.0005630 (PMC13561322; doi:10.1371/journal.pgph.0005630)
Supplement: Supplementary file 11 [file pgph.0005630.s011.docx]

**Predicted choice and acceptability of regimens for tuberculosis preventive therapy among people living with HIV in Uganda – a discrete choice experiment**

**PGPH-D-23-03555**

**Review comments:**

This manuscript presents the results of a discrete choice experiment (DCE) to explore preference structures of participants sampled from the Mulago Immune Suppression Syndrome clinic in Kampala, Uganda regarding different tuberculosis preventive therapy (TPT) regimens. The study aimed to understand which TPT regimen characteristics were most important to people living with HIV when choosing between different regimens either currently available in Uganda or potentially part of the scale up of TPT programme.

The manuscript is well written and the discussion of the findings is clear. However, the description of the methods could benefit from additional detail to improve clarity and transparency. The approach to the statistical analysis needs to be refined to better align with the stated objectives — there are several comments and recommendations below about how this can be done. Some of the results appear to be inconsistent or are inadequately explained for the reader to understand what is being presented — again there are several comments which may help to improve clarity.

Overall, I would like to see this manuscript published, but I believe there are several issues that need to be addressed before it can be accepted. I hope the comments below will be helpful in strengthening the paper.

**Comments on the introduction:**

1. The introduction section is well formulated and gives a clear sense of the lay of the land regarding the TPT programme, Globally and in Uganda, and a clear rationale for the study. However, including some additional detail about the scale of the TB and HIV epidemics in Uganda could help to strengthen the articulation of the need for expanded TPT among people living with HIV. Any details about current TPT completion rates in Uganda would also be useful for readers to understand the extent of the problem and the need for the introduction of new regimens.
2. The study objectives are clearly stated. However, I find objective 3 as stated in the introduction to be somewhat misaligned with the approach to the analysis. Objective 3 states: “evaluate associations between patient characteristics and a preference for no TPT”, but the analysis compares patient characteristics of participants who always accept TPT regardless of the regimen attributes with those of participants who sometimes chose no TPT depending on the regimen characteristics of the two choice alternatives in a choice set. I provide a more thorough review of this approach below, but regardless of the final decision of the authors, the formulation of Objective 3 in the introduction should be better aligned with the approach to the analysis in the methods section.
3. I think the manuscript could also be significantly strengthened by including details (perhaps in the form of a table) about the characteristics of each of the current TPT regimens that are available, which ones are used in Uganda (currently or on the horizon). This table could look similar to Table 1, however the details in Table 1 relate to the modelled scenarios, and only appears at the end of the methods section. Including these details early on as they relate to the global and national TPT programme, and to the design of the DCE would help readers who are not intimately familiar with the characteristics of the different regimens to have a clearer sense of the purpose of the study, and introduce the idea that regimen duration might not be the primary differentiating factor between the different regimens that could be rolled out.

**Comments on the Methods and Results:**

4. Is it possible to say something about the statistical efficiency of the design? The methods section states that “each participant completed nine random choice tasks…” but it is difficult to understand what this really means. Is it 9 choice tasks randomly selected from the full factorial design, where each participant sees a unique set of choice sets, or is it 9 randomly selected choice tasks from the full factorial that are seen by all participants? Or was a large fractional factorial design generated and blocked into several versions? Some additional detail here would be useful for readers interested in understanding the robustness of the experimental design. There are a several useful checklists that could help articulate the experimental design characteristics in this regard. For example in a useful user guide by Emily Lancsar and Jordan Louviere (DOI: [10.2165/00019053-200826080-00004](https://doi.org/10.2165/00019053-200826080-00004)), the authors suggest reporting on the following:

- What type of design was used? Full factorial? Fractional factorial? If fractional, which effects are identified: main effects; main effects + higher order interactions?
- How were the profiles generated and allocated to choice sets?
- What are the properties of the design?
- What is the efficiency of the design?
- Was identification checked (e.g. is the variance-co-variance matrix block diagonal)?
- Was the design blocked into versions? If so, how were choice sets allocated to versions? Were the resulting properties of the versions checked?
- Were respondents randomly allocated to versions?
- How many choice sets were considered per respondent?
- If some profiles were implausible – how was implausibility defined and how was it addressed?

More recent checklists are also available and useful as a potential reporting standard (see Ride, J et. al. 2024, DOI: [10.1007/s40273-024-01431-6](https://doi.org/10.1007/s40273-024-01431-6))

5. The design choice to include both a forced choice initially and an opt out option once participants have made the forced choice has the potential to strengthen the analysis and robustness of the results. Forced choice designs are useful because they can maximise the amount of information regarding participants’ willingness to trade off between different attributes and different levels of each attribute. However, they do not provide an anchor for demand, which makes inferences about uptake tenuous at best. Opt out designs, on the other hand provide an anchor for estimates of demand, but information about participants’ willingness to make trade-offs is reduced when participants opt out from making choices — this potentially leads to scarce data biases, especially when participants consistently opt out when particular attribute levels are present.

The approach to the mixed-effects logistic regression modelling in this manuscript therefore appears somewhat odd, as it mixes the objective to assess preference structures with the objective to assess demand and the attribute levels most likely associated with TPT refusal. It appears from the description in the statistical analysis section that for every choice set where the selected regimen was preferred over no treatment, the alternative regimen in the choice set was dropped from the regression. While this is not an explicitly incorrect approach, it results in half of the information for the majority of the choice sets being excluded from the analysis — given that this was the case for the large majority of the choice sets in the experiment, this approach disregards the strength of the DCE methodology to analyse preference structures and evaluate trade-offs between attributes and level in favour of estimating the potential cut-off point for regimen characteristics that are unambiguously preferable to no treatment. Further, it appears that in cases where participants opted out, both alternatives in the choice set were categorised as “unacceptable”, which means information about what attributes and levels are driving the unacceptability in relation to each other is also lost.

Given that the simulation analysis already explores the preference for TPT over no TPT, a potentially more useful approach to the regression analysis would be to separate out the investigation of the two objectives by treating the forced choice and opt out designs separately. This would mean first conducting the regression analysis as a forced choice design to understand preference structures and drivers of choice, by using a binary mixed-effects model that disregards the additional information about whether either of the alternatives would be acceptable or not. This would result in far more data being included in the analysis with more robust results about what trade-offs participants would be willing to make between attributes and levels and potentially reducing any sparse data bias. These results would provide a clearer picture about which attributes are most important and which levels are most preferred. The second analysis using the current approach could then be integrated with the simulation modelling (which essentially are trying to respond to the same objective to predict TPT over no TPT) to explain what the preference structures mean in relation to the different available (and potentially available) regimens in Uganda. This approach would maximise the value of both forced choice and opt out designs in this study.

In the current presentation of

6. Part of the value of using mixed-effects logistic regression is the estimation of standard deviations, which help to understand preference heterogeneity among the participants. These results are not reported in the manuscript, and could be useful for readers to understand the consistency of the findings across participants.
7. The manuscript explains the approach to addressing the potential sparse data bias in the logistic regression by excluding some attributes and by combining the levels for other attributes in the analysis. This raises two important questions: 1) is this an appropriate approach to addressing this potential source of bias; and 2) is the way in which attribute levels have been combined appropriate in a non-statistical sense?

The first question is relatively easily addressed, either by using the approach to the logistic regression described above (separating out the forced choice analysis from the demand analysis, which may result in more robust estimates given the dramatic increase in the amount of data in the model about preference structures and trade-offs), or by referencing the appropriate literature (or both).

To address the second question, it may be necessary to provide additional justification as to how the attribute levels have been combined. For example, the categories for 1 pill and 5 pills have been combined and compared to 10 pills for statistical reasons, but none of the regimens used in the simulation analysis (in Table 1) have more than 4 pills per dose. So all of the information to understand preferences between 1 and 4 pills is lost in the regression analysis using this approach and raises questions about how the Shares of Preference Model is treating the scarce data bias (and whether the findings from the regression and simulation are comparable). It is not clear from the explanation of the simulation analysis in the methods section, but it appears that the same data manipulation (combining attribute levels and excluding some attributes) was not necessary for the Shares of Preference Model. This raises questions about the comparability of the results from the two analyses.

**Specific comments about the scenario analysis:**

8. In the scenarios detailed in Table 1, 3HP has a 4 pills per dose, but in the results in Figure 1 3HP references 10 pills. This inconsistency should be addressed.
9. It is unclear whether the percentages presented in the scenario analysis for the predicted choices for existing regimens refer to participants or choice scenarios (e.g., whether 88.1% of participants prefer 6H over no TPT, or whether in 88.1% of the choice scenarios, 6H was preferred over no TPT). This clarification matters because of the large percentage of participants who were willing to accept TPT in every scenario presented to them.
10. The results seem to suggest that the number of pills and whether or not the regimen would require an ART adjustment are the two primary drivers of preferences between regimens. In Figure 1A, I would suggest including information about the ART adjustment in addition to the number of pills in the row labels on the left of the figure to emphasise this point.
11. Figure 1B is particularly useful in showing how participants might switch between regimens if and when new formulations like 3HP and 3HP FDC become available in Uganda. Introducing 3HP in addition to 6H increases acceptability of TPT by only a small percentage of 3.5%, but more significantly shows that nearly 50% of people who would otherwise have accepted 6H would switch to 3HP. Similarly introducing 3HP FDC in addition to 6H could increase acceptability to nearly 100% but would mean that almost all participants would want to switch from 6H to 3HP FDC. This is surely worth including in the discussion section and could be strengthened by giving some thought to what this might mean programmatically (in terms of the economic or financial cost, the burden on the health system, the burden on patients, how this could relate to treatment completion rates based on other available evidence etc.).
12. Related to the point above, is the fact that currently, the drug costs for 6H are significantly lower than 3HP. Given the fact that pill count appears to be one of the most important drivers of choice, a useful addition to the scenario analysis might be to understand the impact on demand/acceptability/non-refusal might be to show what the impact would be of investing in developing and introducing a single pill dose of 6H (compared to the standard 2 pill dose of 6H that was modelled). Often global and national guidelines and recommendations focus on the difference between regimens particularly in terms of the duration of treatment. But the results of this study show that pill count is significantly more important in this population — a unique contribution of a DCE of this nature. Therefore, showing how a single pill dosing of the same regimen could be useful in changing acceptability.
13. The results presented in Figure 2 seem odd, and either need to be revisited or explained in more detail (either in the methods or in the results). If I am reading this correctly, then the analysis using the ideal scenario as a reference effectively shows the decrease in acceptability that should be expected when swapping each level of each attribute in the ideal scenario for the worst level one at a time. The 3HP scenario is not as favourable as the ideal scenario, and so I would expect that systematically swapping the levels of the 3HP scenario for the worst level of each attribute would result in a smaller decrease in acceptability than using the ideas as a reference, but this is not the case. The 3HP reference scenario has bigger degreases in every attribute than the ideal reference scenario. As an example, the results imply that changing the number of pills from 1 pill (the ideal) to 10 pills (the worst) results in a 6% decrease in acceptability, but changing from 4 pills (3HP) to 10 pills results in a 15.4% decrease in acceptability. This finding appears to violate the principle of transitivity, unless there are other details that are missing, for example a different sub-sample, or a different interpretation of the results that is not clearly explained. The result in the 3HP reference scenario in Figure 2 (0.9% of participants prefer no TPT compared to 3HP) is also inconsistent with the result shown in Figure 1B (8.4% of participants prefer no TPT even when 3HP is offered in addition to 6H).
14. The last row in Figure 2 appears to combine mild and moderate side effects into one “AE risk” category. However, the details in the description of the ideal scenario and the 3HP scenario have them separated out. Was this modelled differently, combining these attributes, or is there a row missing from Figure 2? For consistency, it would be good to see a closer alignment between the text and the figure.

**Comments on the analysis of participant characteristics in relation to TPT acceptance or refusal:**

15. Although it is not explicit in the methods or results section, it appears that a binary outcome variable has been generated to explore how participant characteristics relate to TPT acceptance or refusal, where 0=(participants who accepted TPT in every scenario) and 1=(participants who rejected TPT in at least one scenario, including the very few who rejected every scenario). I find this approach to the analysis of how participant characteristics relate to TPT refusal to be misleading for the following reason:

Participants who are willing to accept some regimens but not others are likely doing so because of changes in the regimen characteristics, rather than because of their own personal characteristics, and according to the results in the paper, nearly all would really be willing to accept TPT if a combination of 6H and 3HP were offered in Uganda. This means that the characterisation of these participants as potential TPT refusers is somewhat problematic, and the framing of the results (in the results and discussion sections) that participants “with any education, shorter time on ART, and concurrent medications were more likely to refuse some TPT regimens” might be interpreted by readers to mean that these participants are more likely to refuse TPT entirely. Participants in the “sometimes reject TPT category are likely rejecting some choice sets where both alternatives are “worse” than 6H. But because 6H is currently available in Uganda, it does not seem useful to characterise people who would refuse regimens that are worse than 6H as TPT refusers. Although the current approach to the analysis is not necessarily incorrect, it is somewhat misleading and does not appear to add enough value in its current form to include in the manuscript.

An alternative approach, which could add value if an analysis of participant characteristics is still to be included in the paper (although may not produce significant results), might be to use an outcome variable that reflects the characteristics of the participants who would either accept or reject 6H in the scenario analysis, and a second outcome variable to compare the characteristics of participants who would switch from 6H to 3HP if it was introduced with those who would still prefer 6H.

**Comments on the Discussion:**

16. The discussion is well written and situates the findings of this study within the existing body of knowledge. In particular, the emphasis of the findings from the DCE which disentangle the characteristics of treatment regimens that really matter from the current emphasis on treatment duration in the guidelines is a useful addition to the literature. The discussion about how the introduction of new regimens might affect treatment completion rates is also particularly useful, because increased treatment completion rates will have a significant effect on cost-effectiveness.
17. The strong recommendation in the conclusion that the “…continued rollout [of 3HP] to replace 6H should be prioritised” needs to be grounded more in the discussion section in a discussion point about the impact on the health system. In point 11 and 12 above, I have provided some suggestions about how this discussion point might be formulated, but there are no doubt other approaches to addressing this point. At face value, it would be difficult to argue that 6H (which the scenario analysis shows is acceptable to nearly 90% of participants in this study) should be entirely replaced by 3HP FDC to increase acceptability to nearly 100% if the cost to the system would be too high to bear. A 90% acceptance rate is already extremely high, and it would be unwise to recommend a complete overhaul of the 6H TPT programme without stronger evidence that 3HP FDC would be affordable and significantly improve health outcomes.
