# Supplementary material for: Predicted choice and acceptability of regimens for tuberculosis preventive treatment among people living with HIV in Uganda – A discrete choice experiment
Source: PLOS Glob Public Health. 2026 Sep 10;6(9):e0005630. doi: 10.1371/journal.pgph.0005630 (PMC13561322; doi:10.1371/journal.pgph.0005630)
Supplement: Supplementary file 12 [file pgph.0005630.s012.docx]

**Overall:** Overall, this is a well-conducted and policy-relevant study that contributes important insights into patient preferences for TB preventive treatment among PLHIV. The results strongly support the scale-up of shorter, simpler regimens, particularly 3HP fixed-dose combinations, to improve uptake and person-centred care in high-burden settings. The discrete choice experiment is well-designed and allows quantification of patient preferences. The study aligns with growing emphasis on person-centred TB prevention. However, several areas would benefit from clarification and further discussion, particularly regarding generalizability, interpretation of acceptability results, and implications for programmatic implementation.

Abstract:

1. The first sentence can be improved for better flow, as it currently sounds incomplete. The sentence can be written as “Little is known about people living with HIV’s preferences for different TPT regimens, or the conditions under which they’d accept treatment.”

Setting and participants

1. Please add some detail about the selection and recruitment process for the participants and clarify the time interval between completion of the prior TPT to study participation as this could influence choices.

Procedures

1. Please clarify whether participants received any standardized explanation of regimen attributes prior to completing the survey, and how understanding of the explanations was assessed.

DCE design

1. Line 110: italicise the survey text to highlight that it is directly copied from the survey, and increase readability.
2. Line 118: As above, highlight survey text to increase readability. Secondly, please clarify there is text missing in the quoted segment, as I see use of an ellipsis.

Statistical analysis

1. Please provide a brief explanation of hierarchical Bayesian estimation for readers less familiar with DCE methodology.

Results

1. Line 229: Close bracket after “(Longer time on ART decreased the odds of refusing any regimen (OR=0.72 per 5 years, 95% CI 0.54 – 0.97”

Discussion:

1. A large majority of participants (over 90%) had previously received TPT. This may have influenced both familiarity with TPT and willingness to accept treatment. While the authors acknowledge this limitation, it would strengthen the paper to:

- Provide additional discussion on how prior exposure to TPT might bias preferences
- Clarify whether previous regimen experience (e.g., 6H vs 3HP) influenced responses
- Discuss implications for TPT-naive populations

2. The finding that side effects did not meaningfully influence acceptability is interesting but somewhat counterintuitive. The discussion could be expanded to explore possible explanations, such as:

- Difficulty interpreting probability-based risk descriptions
- Trust in healthcare providers
- Hypothetical framing compared to actual experiences with side effects

3. The study was conducted at a single large urban HIV clinic in Kampala. The discussion should more clearly address how preferences might differ in:

- Rural settings
- Newly diagnosed PLHIV
- Individuals not yet engaged in HIV care

4. The findings support the rollout of 3HP fixed-dose combinations. However, the discussion could expand on the practical implications for TB and HIV programs in Uganda and broadly, such as:

- Counselling approaches when multiple regimens are available
- Implementation of patient choice models in routine care

Linking the findings more explicitly to programmatic decision-making would strengthen the policy relevance.
