# Supplementary material for: Predicted choice and acceptability of regimens for tuberculosis preventive treatment among people living with HIV in Uganda – A discrete choice experiment
Source: PLOS Glob Public Health. 2026 Sep 10;6(9):e0005630. doi: 10.1371/journal.pgph.0005630 (PMC13561322; doi:10.1371/journal.pgph.0005630)
Supplement: Supplementary file 13 [file pgph.0005630.s013.docx]

Dr. Graeme Hoddinott

Academic Editor

PLOS Global Public Health

Dear Dr. Hoddinott,

Please find enclosed our revision for our manuscript entitled “Predicted choice and acceptability of regimens for tuberculosis preventive treatment among people living with HIV in Uganda – a discrete choice experiment”.

We are grateful for your and reviewers’ comments and believe they made the reporting in our manuscript stronger. We thank you for the opportunity to resubmit this work as a research article for PLOS Global Public Health.

Please find our point-by-point responses to comments below. Line numbers refer to the version with tracked version.

We certify that all contributing authors meet the authorship criteria, have reviewed the manuscript, and agree with its contents, and that this submission is original and is not under consideration by any other journal.

We thank you for your consideration of our manuscript and are looking forward to hearing from you.

On behalf of all authors,

Dr. Hélène Aschmann

**Editor:**

1. Please include a complete copy of PLOS’ questionnaire on inclusivity in global research in your revised manuscript. Our policy for research in this area aims to improve transparency in the reporting of research performed outside of researchers’ own country or community. The policy applies to researchers who have travelled to a different country to conduct research, research with Indigenous populations or their lands, and research on cultural artefacts. The questionnaire can also be requested at the journal’s discretion for any other submissions, even if these conditions are not met. Please find more information on the policy and a link to download a blank copy of the questionnaire here: https://journals.plos.org/globalpublichealth/s/best-practices-in-research-reporting. Please upload a completed version of your questionnaire as Supporting Information when you resubmit your manuscript.

**RESPONSE:** We have included the checklist as a supplemental file as instructed.

2. Please amend your detailed Financial Disclosure statement. This is published with the article. It must therefore be completed in full sentences and contain the exact wording you wish to be published.

i. Please clarify all sources of financial support for your study. List the grants, grant numbers, and organizations that funded your study, including funding received from your institution. Please note that suppliers of material support, including research materials, should be recognized in the Acknowledgements section rather than in the Financial Disclosure. 
ii. State the initials, alongside each funding source, of each author to receive each grant. For example: "This work was supported by the National Institutes of Health (####### to AM; ###### to CJ) and the National Science Foundation (###### to AM)."
iii. State what role the funders took in the study. If the funders had no role in your study, please state: “The funders had no role in study design, data collection and analysis, decision to publish, or preparation of the manuscript.”
iv. If any authors received a salary from any of your funders, please state which authors and which funders.

**RESPONSE:** Please excuse this oversight. We have added the funding statement on page 19.

3. In the online submission form, you indicated that “Deidentified individual participant data that support the findings of this study are available from the corresponding author upon reasonable request after approval of a proposal.”.

All PLOS journals now require all data underlying the findings described in their manuscript to be freely available to other researchers, either

1. In a public repository,

2. Within the manuscript itself, or

3. Uploaded as supplementary information.

This policy applies to all data except where public deposition would breach compliance with the protocol approved by your research ethics board. If your data cannot be made publicly available for ethical or legal reasons (e.g., public availability would compromise patient privacy), please explain your reasons by return email and your exemption request will be escalated to the editor for approval. Your exemption request will be handled independently and will not hold up the peer review process, but will need to be resolved should your manuscript be accepted for publication. One of the Editorial team will then be in touch if there are any issues.

**RESPONSE**: We will upload the deidentified data to a public repository (Dryad) upon acceptance and prior to publication. We have uploaded it for peer review and it should be available soon under the following doi: doi.org/10.5061/dryad.31zcrjf34.

4. Some material included in your submission may be copyrighted. According to PLOS’s copyright policy, authors who use figures or other material (e.g., graphics, clipart, maps) from another author or copyright holder must demonstrate or obtain permission to publish this material under the Creative Commons Attribution 4.0 International (CC BY 4.0) License used by PLOS journals. Please closely review the details of PLOS’s copyright requirements here: PLOS Licenses and Copyright. If you need to request permissions from a copyright holder, you may use PLOS's Copyright Content Permission form.

Please respond directly to this email or email the journal office and provide any known details concerning your material's license terms and permissions required for reuse, even if you have not yet obtained copyright permissions or are unsure of your material's copyright compatibility.

Potential Copyright Issues:

a. Figure S1: Please confirm whether you drew the images / clip-art within the figure panels by hand. If you did not draw the images, please provide (a) a link to the source of the images or icons and their license / terms of use; or (b) written permission from the copyright holder to publish the images or icons under our CC-BY 4.0 license. Alternatively, you may replace the images with open source alternatives. See these open source resources you may use to replace images / clip-art:

- https://commons.wikimedia.org

- <https://openclipart.org/>

**RESPONSE**: Thank you for the opportunity to clarify. Our team drew the images in Figure S1 by hand. We have previously published them in another manuscript.

**Reviewer #1:**

5. This manuscript and the findings from you study are important additions to the literature. However, I believe that the manuscript needs to be strengthened, and the approach to the analysis reconsidered before the paper can be published. I have provided detailed feedback to help strengthen the manuscript and refine the approach to the data analysis for your consideration, attached. I hope that this review will be useful for strengthening the manuscript and the implications of your work for policy, guidelines and practice.

This manuscript presents the results of a discrete choice experiment (DCE) to explore preference structures of participants sampled from the Mulago Immune Suppression Syndrome clinic in Kampala, Uganda regarding different tuberculosis preventive therapy (TPT) regimens. The study aimed to understand which TPT regimen characteristics were most important to people living with HIV when choosing between different regimens either currently available in Uganda or potentially part of the scale up of TPT programme.

The manuscript is well written and the discussion of the findings is clear. However, the description of the methods could benefit from additional detail to improve clarity and transparency. The approach to the statistical analysis needs to be refined to better align with the stated objectives — there are several comments and recommendations below about how this can be done. Some of the results appear to be inconsistent or are inadequately explained for the reader to understand what is being presented — again there are several comments which may help to improve clarity.

Overall, I would like to see this manuscript published, but I believe there are several issues that need to be addressed before it can be accepted. I hope the comments below will be helpful in strengthening the paper.

**RESPONSE:** Thank you for your thoughtful and detailed review. We address your comments and recommendations below and are grateful for the chance to improve this manuscript.

6. The introduction section is well formulated and gives a clear sense of the lay of the land regarding the TPT programme, Globally and in Uganda, and a clear rationale for the study. However, including some additional detail about the scale of the TB and HIV epidemics in Uganda could help to strengthen the articulation of the need for expanded TPT among people living with HIV. Any details about current TPT completion rates in Uganda would also be useful for readers to understand the extent of the problem and the need for the introduction of new regimens.

**RESPONSE:** Thank you for your suggestion to strengthen the introduction. We have added the following to provide further context on TB in TPT completion among PLHIV in Uganda (l.65-71):

“*While published TPT completion rates among PLHIV in Uganda are typically above 90%[7,8], completion in actual practice is believed to be substantially lower. For example, completion of isoniazid preventive therapy in northeastern Uganda was reported to be 32%, with over half of non-completions due to drug stockouts.[9]* Person-centered strategies remain necessary to ensure high coverage among people newly enrolled in HIV care in Uganda [2]. *In 2024, of the estimated 99 000 incident TB cases in Uganda, 33% were among PLHIV [2].*”

In addition, note that internationally, studies report that only about half of those initiating the 6H regimen complete treatment (Alsdurf et al. 2016 [doi.org/10.1016/S1473-3099(16)30216-X](https://doi.org/10.1016/S1473-3099(16)30216-X), Melgar et al. 2020 [dx.doi.org/10.15585/mmwr.mm6912a3](http://dx.doi.org/10.15585/mmwr.mm6912a3)).

7. The study objectives are clearly stated. However, I find objective 3 as stated in the introduction to be somewhat misaligned with the approach to the analysis. Objective 3 states: “evaluate associations between patient characteristics and a preference for no TPT”, but the analysis compares patient characteristics of participants who always accept TPT regardless of the regimen attributes with those of participants who sometimes chose no TPT depending on the regimen characteristics of the two choice alternatives in a choice set. I provide a more thorough review of this approach below, but regardless of the final decision of the authors, the formulation of Objective 3 in the introduction should be better aligned with the approach to the analysis in the methods section.

**RESPONSE:** Thank you for catching this inconsistency. Based on your further comment below on this analysis, we have elected to remove this objective and analysis from this manuscript. We have previously reported preference heterogeneity in this study population and believe this analysis does not add significant insight beyond that already reported ([doi.org/10.1002/jia2.26390](https://doi.org/10.1002/jia2.26390)).

8. I think the manuscript could also be significantly strengthened by including details (perhaps in the form of a table) about the characteristics of each of the current TPT regimens that are available, which ones are used in Uganda (currently or on the horizon). This table could look similar to Table 1, however the details in Table 1 relate to the modelled scenarios, and only appears at the end of the methods section. Including these details early on as they relate to the global and national TPT programme, and to the design of the DCE would help readers who are not intimately familiar with the characteristics of the different regimens to have a clearer sense of the purpose of the study, and introduce the idea that regimen duration might not be the primary differentiating factor between the different regimens that could be rolled out.

**RESPONSE:** Thank you for your helpful suggestion. We have added a column to Table 1 to clarify which regimens are currently available in Uganda to people living with HIV. We have moved this table to the beginning of the methods section, and the local availability of regimens is a valuable addition to clarify context for the simulations performed, in particular the ones that follow historic availability.

**Comments on the Methods and Results:**

9. Is it possible to say something about the statistical efficiency of the design? The methods section states that “each participant completed nine random choice tasks…” but it is difficult to understand what this really means. Is it 9 choice tasks randomly selected from the full factorial design, where each participant sees a unique set of choice sets, or is it 9 randomly selected choice tasks from the full factorial that are seen by all participants? Or was a large fractional factorial design generated and blocked into several versions? Some additional detail here would be useful for readers interested in understanding the robustness of the experimental design. There are a several useful checklists that could help articulate the experimental design characteristics in this regard. For example in a useful user guide by Emily Lancsar and Jordan Louviere (DOI: [10.2165/00019053-200826080-00004](https://doi.org/10.2165/00019053-200826080-00004)), the authors suggest reporting on the following:

- What type of design was used? Full factorial? Fractional factorial? If fractional, which effects are identified: main effects; main effects + higher order interactions?
- How were the profiles generated and allocated to choice sets?
- What are the properties of the design?
- What is the efficiency of the design?
- Was identification checked (e.g. is the variance-co-variance matrix block diagonal)?
- Was the design blocked into versions? If so, how were choice sets allocated to versions? Were the resulting properties of the versions checked?
- Were respondents randomly allocated to versions?
- How many choice sets were considered per respondent?
- If some profiles were implausible – how was implausibility defined and how was it addressed?

More recent checklists are also available and useful as a potential reporting standard (see Ride, J et. al. 2024, DOI: [10.1007/s40273-024-01431-6](https://doi.org/10.1007/s40273-024-01431-6))

**RESPONSE**: Thank you for the opportunity to clarify the design and for the reminder about the reporting checklist, which was not yet available when we published the original manuscript on this discrete choice experiment. We have carefully reviewed the checklist and confirm all the required information is included in the original manuscript, which is cited in this simulation study ([doi.org/10.1002/jia2.26390](https://doi.org/10.1002/jia2.26390)). In addition, we have added the following details to this manuscript to fulfill the checklist (l.135-137):

“Participants were randomly allocated to one of 500 randomly generated sets of the nine random choice tasks, using the “balanced overlap” method to allow for analysis of interaction terms.”

To further clarify the questions you list above, the “balanced overlap” design maximizes statistical design efficiency but also creates some overlap (i.e., the two profiles in the choice set have the same level for an attribute). This helps quantify preferences even when one attribute is dominant (i.e., far more important) for a respondent. There was no restriction for implausible combinations (e.g., a 1 month weekly treatment was allowed, as future regimens may still further reduce the total number of doses). Participants were randomly allocated to one of 500 versions, with 9 random choice tasks each. In addition, as described in the manuscript, they all completed two fixed choice tasks (one dominant task to check comprehension and one that approximated 1HP vs 3HP). The statistical efficiency is usually only relevant and reported when different designs are evaluated and compared. We kept the description of the design in this simulation study as brief as possible, as we feel it is more appropriate for the scope of this manuscript. However, if preferred by the Editor, we can add additional details to a methodological appendix.

10. The design choice to include both a forced choice initially and an opt out option once participants have made the forced choice has the potential to strengthen the analysis and robustness of the results. Forced choice designs are useful because they can maximise the amount of information regarding participants’ willingness to trade off between different attributes and different levels of each attribute. However, they do not provide an anchor for demand, which makes inferences about uptake tenuous at best. Opt out designs, on the other hand provide an anchor for estimates of demand, but information about participants’ willingness to make trade-offs is reduced when participants opt out from making choices — this potentially leads to scarce data biases, especially when participants consistently opt out when particular attribute levels are present.

The approach to the mixed-effects logistic regression modelling in this manuscript therefore appears somewhat odd, as it mixes the objective to assess preference structures with the objective to assess demand and the attribute levels most likely associated with TPT refusal. It appears from the description in the statistical analysis section that for every choice set where the selected regimen was preferred over no treatment, the alternative regimen in the choice set was dropped from the regression. While this is not an explicitly incorrect approach, it results in half of the information for the majority of the choice sets being excluded from the analysis — given that this was the case for the large majority of the choice sets in the experiment, this approach disregards the strength of the DCE methodology to analyse preference structures and evaluate trade-offs between attributes and level in favour of estimating the potential cut-off point for regimen characteristics that are unambiguously preferable to no treatment. Further, it appears that in cases where participants opted out, both alternatives in the choice set were categorised as “unacceptable”, which means information about what attributes and levels are driving the unacceptability in relation to each other is also lost.

Given that the simulation analysis already explores the preference for TPT over no TPT, a potentially more useful approach to the regression analysis would be to separate out the investigation of the two objectives by treating the forced choice and opt out designs separately. This would mean first conducting the regression analysis as a forced choice design to understand preference structures and drivers of choice, by using a binary mixed-effects model that disregards the additional information about whether either of the alternatives would be acceptable or not. This would result in far more data being included in the analysis with more robust results about what trade-offs participants would be willing to make between attributes and levels and potentially reducing any sparse data bias. These results would provide a clearer picture about which attributes are most important and which levels are most preferred. The second analysis using the current approach could then be integrated with the simulation modelling (which essentially are trying to respond to the same objective to predict TPT over no TPT) to explain what the preference structures mean in relation to the different available (and potentially available) regimens in Uganda. This approach would maximise the value of both forced choice and opt out designs in this study.

In the current presentation of

**RESPONSE**: Thank you for this thoughtful discussion of our analysis approach. We agree with you that the simulation analysis already explores the preference for TPT over no TPT (on an absolute scale), including the effect of varying individual levels and attributes. Please note that we have already reported the results using the full range of preference data in a prior manuscript ([doi.org/10.1002/jia2.26390](https://doi.org/10.1002/jia2.26390)). To ensure future readers do not miss this, we have added the following sentence at the end of the introduction section (l.87-89):

**“**We have previously reported the main effects and heterogeneity of preferences measured in this discrete choice experiment [13], and aim to provide further insights into the impact of regimen characteristics on the acceptability of TPT in this simulation study.”

To address your concern, we have moved the regression analysis to the supplementary material. We believe that it can give further insights into the (relative) effects of individual attributes. Furthermore, the fact that it uses a different analytic approach and a more restricted data set compared to the preference simulation (and the findings are in full agreement) demonstrates the robustness of our results, in the spirit of a sensitivity analysis. Yet, as you discuss below, the regression analysis has some limitations, and its results may be more appropriately less emphasized in the main manuscript.

11. Part of the value of using mixed-effects logistic regression is the estimation of standard deviations, which help to understand preference heterogeneity among the participants. These results are not reported in the manuscript, and could be useful for readers to understand the consistency of the findings across participants.

**RESPONSE**: Thank you for this suggestion. To address the heterogeneity across participants, we have added the standard deviation of the random intercept for participants (SD = 4.57) to Table S6. In addition, we have added the logOR (i.e., beta coefficients) for the fixed effects together with the standard errors to Table S6.

**Table S6**: Regimen features associated with unacceptable vs acceptable TPT regimens. The analysis was conducted using mixed effects logistic regression.

|  | aOR | [95% CI] | logOR | SE |
| --- | --- | --- | --- | --- |
| Intercept | - | - | -9.89 | 0.81 |
| Duration |  |  |  |  |
| 3 or 6 months vs 1 month | 4.9 | [3.5, 6.8] | 1.58 | 0.17 |
| Dosing frequency |  |  |  |  |
| More than weekly vs weekly | 3.4 | [2.5, 4.6] | 1.22 | 0.16 |
| Number of pills per dose |  |  |  |  |
| 10 vs 1 or 5 | 24.5 | [16.6, 36.3] | 3.20 | 0.20 |
| ART dosage adjustment vs none | 27.4 | [18.5, 40.7] | 3.31 | 0.20 |
|  |  |  |  | **SD** |
| Random effects (participants) |  |  |  | 4.57 |

ART: antiretroviral therapy, aOR: adjusted odds ratio, logOR: log odds ratio or beta coefficients, SD: standard deviation, SE: standard error

We have kept the remaining regression reporting (Tables S7 and S8) focused on the adjusted odds ratios and confidence intervals, consistent with their role as supporting analyses.

12. The manuscript explains the approach to addressing the potential sparse data bias in the logistic regression by excluding some attributes and by combining the levels for other attributes in the analysis. This raises two important questions: 1) is this an appropriate approach to addressing this potential source of bias; and 2) is the way in which attribute levels have been combined appropriate in a non-statistical sense?

The first question is relatively easily addressed, either by using the approach to the logistic regression described above (separating out the forced choice analysis from the demand analysis, which may result in more robust estimates given the dramatic increase in the amount of data in the model about preference structures and trade-offs), or by referencing the appropriate literature (or both).

To address the second question, it may be necessary to provide additional justification as to how the attribute levels have been combined. For example, the categories for 1 pill and 5 pills have been combined and compared to 10 pills for statistical reasons, but none of the regimens used in the simulation analysis (in Table 1) have more than 4 pills per dose. So all of the information to understand preferences between 1 and 4 pills is lost in the regression analysis using this approach and raises questions about how the Shares of Preference Model is treating the scarce data bias (and whether the findings from the regression and simulation are comparable). It is not clear from the explanation of the simulation analysis in the methods section, but it appears that the same data manipulation (combining attribute levels and excluding some attributes) was not necessary for the Shares of Preference Model. This raises questions about the comparability of the results from the two analyses.

**RESPONSE**: We apologize for the confusion about the importance of 10 pills, which was not appropriately explained in Table 1. In our view, 10 pills is an important comparator because 3HP is usually a 10-pill regimen, unless it is provided as a fixed-dose combination. It was important to highlight to decision-makers that rolling out the fixed-dose combination of 3HP should be prioritized. We have added a row in **Table 1** to distinguish between the 3HP “multiple tablet” version and the fixed-dose combination (FDC).

Please also note that the preference between 1 and 5 pills is demonstrated in the sensitivity analysis in Table S8 (previously called Table S7): the odds of refusing TPT are approximately 5 times increased with 5 pills vs 1 pill (aOR 4.7 [95%CI 3.1, 7.1]). This estimate is stable – it is the comparison of 1 vs 10 pills that becomes unstable due to sparse data bias (in this sensitivity analysis, the confidence interval extends from aOR 50.1 to 157.0). We are confident that this approach of combining levels is a valid way to address sparse data bias in statistical terms, and the different versions of the regression (in Tables S6-S8) help to disentangle the effects of the individual levels.

As discussed above, although your suggestion to perform the regression using the full preference data is valid, we have already published these results in a previous paper.

We feel that this regression analysis adds important insights. However, since multiple tables are needed to really understand the effects of the individual levels (e.g., 5 pills and 10 pills), we feel that it is more appropriate to emphasize the simulation results (Figure 2) and move all regression-related results to the supplementary material. We opted not to delete the regression entirely, as we feel that it is a strength of this analysis that (despite the different analytic approaches) it confirms the results of Figure 2 (i.e., ART dosage adjustments and 10 pills have the largest effects on the acceptability of a regimen).

**Specific comments about the scenario analysis:**

13. In the scenarios detailed in Table 1, 3HP has a 4 pills per dose, but in the results in Figure 1 3HP references 10 pills. This inconsistency should be addressed.

**RESPONSE**: Thank you for the opportunity to clarify. It was not adequately contextualized in Table 1 that 3HP is available in two versions, as a “multiple tablet” version (10 pills per dose) and a fixed-dose combination (4 pills per dose). We have added a row in Table 1 to distinguish between the 3HP “multiple tablet” version and the fixed-dose combination (FDC).

**Table 1**: Simulation parameters of TPT regimens for choice predictions.
1HP: 1 month of daily isoniazid-rifapentine, 3HP: 3 months of weekly isoniazid-rifapentine, 3HR: 3 months of daily isoniazid-rifampin, 4R: 4 months of daily rifampin, 6H: 6 months of daily isoniazid, ART: Antiretroviral therapy, FDC: fixed-dose combination, TPT: Tuberculosis preventive treatment

| TPT | Duration  [months] | Frequency | Pills per dose^a^ | Adjust ART dosage | | Mild side effects [%]^c^ | Moderate side effects [%]^c^ | Local availability |
| --- | --- | --- | --- | --- | --- | --- | --- | --- |
| 1HP | 1 | Daily | 4 | | Yes^b^ | 57.0 | 16.8 | No^d^ |
| 3HP FDC | 3 | Weekly | 4 | | No | 11.5 | 6.0 | Yes |
| 3HP | 3 | Weekly | 10 | | No | 11.5 | 6.0 | Yes |
| 3HR | 3 | Daily | 4 | | Yes | 29.7 | 2.3 | No |
| 4R | 4 | Daily | 1 | | Yes | 20.0 | 1.7 | No |
| 6H | 6 | Daily | 2 | | No | 36.1 | 8.2 | Yes |

^a^ Pills per dose includes recommended pills of vitamin B6 if applicable. ^b^ As definitive studies are lacking, we also performed sensitivity analyses where 1HP was assumed to require no ART dosage adjustment. ^c^ Mild side effects were described as transient nausea, dizziness, fatigue, or skin rashes, for about a week, resolving on their own. Moderate or severe side effects were described as bothersome symptoms like tingling feet, allergy, or liver damage, requiring additional visits to the health care facility and which may require stopping medicine or being admitted to the hospital. ^d^ 1HP is recommended in Ugandan guidelines for people living with HIV but not currently available.

14. It is unclear whether the percentages presented in the scenario analysis for the predicted choices for existing regimens refer to participants or choice scenarios (e.g., whether 88.1% of participants prefer 6H over no TPT, or whether in 88.1% of the choice scenarios, 6H was preferred over no TPT). This clarification matters because of the large percentage of participants who were willing to accept TPT in every scenario presented to them.

**RESPONSE**: Thank you for pointing out that this was unclear. The percentages indicate that 88.1% of participants are predicted to prefer 6H over no TPT. A strength of our DCE was that it collected sufficient choice data per participant, enabling us to simulate the preferences of all individuals.

We have added this clarification in the abstract (l.45-46):

“Choice simulations showed that if only 6 months of daily isoniazid (6H) was available, 11.9% *of participants* *were predicted to* prefer no TPT.”

We have also added it in the results section (l.221-222):

“The standard 6H regimen was *predicted to be* preferred over no TPT by 88.1% *of participants*.”

In other instances in the results section, this already seemed clearly explained, for example (l.219-221, l.225-226):

“When comparing the shares of preference for a choice between single existing regimens and no TPT, 3HR, 4R, and 1HP (if requiring ART adjustment) were unacceptable to a large proportion (range, 29-41%) of participants (**Figure 1A**).”

“… adding 3HP FDC to 6H reduced no-TPT preference to 1.2%, with 94.5% of participants predicted to choose 3HP (**Figure 1B**).”

15. The results seem to suggest that the number of pills and whether or not the regimen would require an ART adjustment are the two primary drivers of preferences between regimens. In Figure 1A, I would suggest including information about the ART adjustment in addition to the number of pills in the row labels on the left of the figure to emphasise this point.

**RESPONSE**: Thank you for this suggestion, we have added the suggested labels in Figure 1A, as displayed below.

16. Figure 1B is particularly useful in showing how participants might switch between regimens if and when new formulations like 3HP and 3HP FDC become available in Uganda. Introducing 3HP in addition to 6H increases acceptability of TPT by only a small percentage of 3.5%, but more significantly shows that nearly 50% of people who would otherwise have accepted 6H would switch to 3HP. Similarly introducing 3HP FDC in addition to 6H could increase acceptability to nearly 100% but would mean that almost all participants would want to switch from 6H to 3HP FDC. This is surely worth including in the discussion section and could be strengthened by giving some thought to what this might mean programmatically (in terms of the economic or financial cost, the burden on the health system, the burden on patients, how this could relate to treatment completion rates based on other available evidence etc.).

**RESPONSE:** Thank you for pointing out that this finding could be better emphasized and discussed. We have included the following text in the discussion (l.347-356):

“*More importantly, offering a choice would improve person-centeredness of TB prevention and likely almost entirely displace the 6H regimen**. Notably, if offered 3HP as FDC formulation or 6H, about 84% of individuals would switch from 6H to 3HP.* In addition, our findings showed that 25% of those who would accept 3HP would prefer 1HP (with no ART dosage adjustment) if available, demonstrating that providing TPT regimen choices better aligns with individuals' preferences. *These findings have two important consequences from a public health perspective: offering a choice of short-course regimens may increase cost but also increase TPT coverage more strongly than our simulations suggest. Although 3HP is more expensive that 6H, cost-effectiveness analyses suggest that, at current 3HP pricing, 3HP is likely to be cost-effective relative to 6H due to higher completion [36]*.

17. Related to the point above, is the fact that currently, the drug costs for 6H are significantly lower than 3HP. Given the fact that pill count appears to be one of the most important drivers of choice, a useful addition to the scenario analysis might be to understand the impact on demand/acceptability/non-refusal might be to show what the impact would be of investing in developing and introducing a single pill dose of 6H (compared to the standard 2 pill dose of 6H that was modelled). Often global and national guidelines and recommendations focus on the difference between regimens particularly in terms of the duration of treatment. But the results of this study show that pill count is significantly more important in this population — a unique contribution of a DCE of this nature. Therefore, showing how a single pill dosing of the same regimen could be useful in changing acceptability.

**RESPONSE:** Thank you for this suggestion. To clarify, we modeled the 6H regimen as 2 pills per dose because the tablet of isoniazid is supposed to be taken with a tablet of vitamin B6. We have added this clarification to **Table 1**.

Therefore, the reviewer’s suggestion would be to investigate whether a fixed-dose combination of isoniazid with vitamin B6 should be developed. Despite decades of availability, 6H has not resulted in a meaningful uptake of TPT, nor motivated the development of a fixed-dose combination of isoniazid and vitamin B6. Thus, the current Ugandan guidelines also recommend 3HP as an alternative. Since 3HP is recommended by local guidelines and is widely preferred to 6H, it seems unlikely that innovations to the six-month-long daily regimen would make a difference in actual practice. We would like to avoid confusing readers by making such a suggestion.

While our preference simulations indicate that almost 90% would be willing to take 6H, this reflects the proportion predicted to start treatment and does not reflect the proportion who would likely complete this regimen. As mentioned above, 6H likely has only a completion rate of about 30-50% across settings. This may, in part, be due to more frequent side effects, as research shows that individuals discontinue TPT when side effects interfere with their gainful employment or daily activities.

18. The results presented in Figure 2 seem odd, and either need to be revisited or explained in more detail (either in the methods or in the results). If I am reading this correctly, then the analysis using the ideal scenario as a reference effectively shows the decrease in acceptability that should be expected when swapping each level of each attribute in the ideal scenario for the worst level one at a time. The 3HP scenario is not as favourable as the ideal scenario, and so I would expect that systematically swapping the levels of the 3HP scenario for the worst level of each attribute would result in a smaller decrease in acceptability than using the ideas as a reference, but this is not the case. The 3HP reference scenario has bigger degreases in every attribute than the ideal reference scenario. As an example, the results imply that changing the number of pills from 1 pill (the ideal) to 10 pills (the worst) results in a 6% decrease in acceptability, but changing from 4 pills (3HP) to 10 pills results in a 15.4% decrease in acceptability. This finding appears to violate the principle of transitivity, unless there are other details that are missing, for example a different sub-sample, or a different interpretation of the results that is not clearly explained. The result in the 3HP reference scenario in Figure 2 (0.9% of participants prefer no TPT compared to 3HP) is also inconsistent with the result shown in Figure 1B (8.4% of participants prefer no TPT even when 3HP is offered in addition to 6H).

**RESPONSE:** Thank you for this careful observation. This is indeed correct, the effect of a less preferred level more strongly reduces the acceptability in the presence of other less preferred levels. We believe that the non-transitivity of these effects makes intuitive sense – when people are given the option of a near-ideal regimen, they may be willing to accept even large compromises on a single attribute. But when faced with a regimen that is already less than ideal in many attributes, their tolerance for further compromises is substantially less. We have revised the beginning of our discussion section accordingly (l.292-295):

**“**In this simulation study using preference data from PLHIV in Uganda, we found that TPT with ART dosage adjustments and high pill burden was often unacceptable to PLHIV, particularly *in the context of regimens that contain* other less desirable features like longer durations and daily dosing frequency.”

To clarify Figure 2, we have re-labeled the reference “3HP” more accurately to “3HP FDC”. Note that the 8.4% of participants who would prefer no TPT was if they were offered only the 10-pill 3HP regimen. Figures 1A and 2 show the same result: 99.1% prefer 3HP FDC over no treatment. However, it is true that there is a surprising effect in Figure 1B of adding 6H as a choice, where 1.2% [95% confidence interval: 0.5%, 1.9%] are predicted to prefer no TPT when given the choice of 3HP FDC and 6H, i.e., more than the 0.9% [95%CI: 0.2%, 1.6%] predicted with only 3HP FDC offered. This is within the range of statistical uncertainty in our findings.

19. The last row in Figure 2 appears to combine mild and moderate side effects into one “AE risk” category. However, the details in the description of the ideal scenario and the 3HP scenario have them separated out. Was this modelled differently, combining these attributes, or is there a row missing from Figure 2? For consistency, it would be good to see a closer alignment between the text and the figure.

**RESPONSE:** Thank you for pointing out that this was not adequately explained. Indeed, we modeled the effect of increasing both the frequency of mild and moderate or severe side effects in the last row, as the effects were so small that we believed it did not make sense to show them separately. The main conclusion is that the risk of TPT-related side effects (mild or moderate/severe) did not meaningfully impact our participants’ choices.

We have rephrased the corresponding text for clarity as to how we defined high risk of side effects (l.266):

“… or had a high risk of side effects (90% for mild side effects *and* 20% for moderate or severe side effects).”

In addition, we revised the figure caption:

“Figure 2: Predicted preference of regimens incorporating least preferred attribute levels in A) an ideal TPT regimen with otherwise optimal features (1 pill per week for 1 month, no ART adjustment, 10% risk of mild side effects, and 1% risk of moderate or severe side effects) and B) in a hypothetical TPT regimen that otherwise mirrors 3HP as FDC (4 pills per week for 3 months, no ART adjustment, 11.5% risk of mild side effects, and 6% risk of moderate or severe side effects). In the last row, “high AE risk”, the frequency of mild side effects was increased to 90% and moderate or severe side effects to 20%.

3HP: 3 months of weekly isoniazid and rifapentine, AE: adverse event, ART: antiretroviral therapy, FDC: fixed-dose combination, TPT: tuberculosis preventive therapy”

**Comments on the analysis of participant characteristics in relation to TPT acceptance or refusal:**

20. Although it is not explicit in the methods or results section, it appears that a binary outcome variable has been generated to explore how participant characteristics relate to TPT acceptance or refusal, where 0=(participants who accepted TPT in every scenario) and 1=(participants who rejected TPT in at least one scenario, including the very few who rejected every scenario). I find this approach to the analysis of how participant characteristics relate to TPT refusal to be misleading for the following reason:

Participants who are willing to accept some regimens but not others are likely doing so because of changes in the regimen characteristics, rather than because of their own personal characteristics, and according to the results in the paper, nearly all would really be willing to accept TPT if a combination of 6H and 3HP were offered in Uganda. This means that the characterisation of these participants as potential TPT refusers is somewhat problematic, and the framing of the results (in the results and discussion sections) that participants “with any education, shorter time on ART, and concurrent medications were more likely to refuse some TPT regimens” might be interpreted by readers to mean that these participants are more likely to refuse TPT entirely. Participants in the “sometimes reject TPT category are likely rejecting some choice sets where both alternatives are “worse” than 6H. But because 6H is currently available in Uganda, it does not seem useful to characterise people who would refuse regimens that are worse than 6H as TPT refusers. Although the current approach to the analysis is not necessarily incorrect, it is somewhat misleading and does not appear to add enough value in its current form to include in the manuscript.

An alternative approach, which could add value if an analysis of participant characteristics is still to be included in the paper (although may not produce significant results), might be to use an outcome variable that reflects the characteristics of the participants who would either accept or reject 6H in the scenario analysis, and a second outcome variable to compare the characteristics of participants who would switch from 6H to 3HP if it was introduced with those who would still prefer 6H.

**RESPONSE:** Thank you for your thoughtful discussion of this analysis. As mentioned above, we have decided to follow your advice and remove it from the manuscript. We agree that the largest effect, due to education, most likely means that these participants understood the tasks better, were more discerning, and/or were less affected by social desirability bias. Although we mentioned this in the discussion section, it is possible that readers could misinterpret the analysis if they only read the results. In addition, as our previous manuscript showed, shorter time on ART and concurrent medications were associated with a stronger preference to avoid ART dosage adjustments, so we feel this analysis adds little additional insight.

Although your proposed analyses are insightful, they are unlikely to be sufficiently powered. Therefore, we prefer not to include any new analyses of baseline characteristics and instead refer readers to our other manuscript.

**Comments on the Discussion:**

21. The discussion is well written and situates the findings of this study within the existing body of knowledge. In particular, the emphasis of the findings from the DCE which disentangle the characteristics of treatment regimens that really matter from the current emphasis on treatment duration in the guidelines is a useful addition to the literature. The discussion about how the introduction of new regimens might affect treatment completion rates is also particularly useful, because increased treatment completion rates will have a significant effect on cost-effectiveness.

**RESPONSE:** Thank you.

22. The strong recommendation in the conclusion that the “…continued rollout [of 3HP] to replace 6H should be prioritised” needs to be grounded more in the discussion section in a discussion point about the impact on the health system. In point 11 and 12 above, I have provided some suggestions about how this discussion point might be formulated, but there are no doubt other approaches to addressing this point. At face value, it would be difficult to argue that 6H (which the scenario analysis shows is acceptable to nearly 90% of participants in this study) should be entirely replaced by 3HP FDC to increase acceptability to nearly 100% if the cost to the system would be too high to bear. A 90% acceptance rate is already extremely high, and it would be unwise to recommend a complete overhaul of the 6H TPT programme without stronger evidence that 3HP FDC would be affordable and significantly improve health outcomes.

**RESPONSE:** Thank you for raising this point. We would note that current Ugandan guidelines do recommend 3HP as a TPT option, and that 3HP is generally offered first where resources allow; thus, our argument is more for identifying additional resources to support the policy recommendations that Uganda is already pursuing, rather than overturning existing policy or practice (and we agree that 6H should continue to be offered where 3HP is unavailable or unaffordable to the health system.) Our study did not assess the health system costs or predict completion of any given regimen, though as noted in our revised introduction, 3HP completion generally substantially higher than 6H. We have accordingly refined the conclusion: we still prioritize continued rollout of 3HP FDC, but now on the grounds that it was highly preferred by participants and would increase patient-centeredness, and we have removed the language that it should "replace 6H." Finally, because TPT coverage is not only a consequence of which regimen people would choose, but also delivery strategies, completion rates, and other factors, we revised the final sentence to (l.398-400):

“Future research should explore the feasibility of offering and delivering regimen choices, especially as new TPT options with preferred features become available.”

**Reviewer #2:**

23. Overall, this is a well-conducted and policy-relevant study that contributes important insights into patient preferences for TB preventive treatment among PLHIV. The results strongly support the scale-up of shorter, simpler regimens, particularly 3HP fixed-dose combinations, to improve uptake and person-centred care in high-burden settings. The discrete choice experiment is well-designed and allows quantification of patient preferences. The study aligns with growing emphasis on person-centred TB prevention. However, several areas would benefit from clarification and further discussion, particularly regarding generalizability, interpretation of acceptability results, and implications for programmatic implementation.

**RESPONSE:** Thank you for your detailed and thoughtful review. We appreciate your feedback and the opportunity to improve this manuscript.

24. Abstract:

The first sentence can be improved for better flow, as it currently sounds incomplete. The sentence can be written as “Little is known about people living with HIV’s preferences for different TPT regimens, or the conditions under which they’d accept treatment.”

**RESPONSE:** Thank you, we have revised the sentence as you suggested (l.30-32).

25. Setting and participants

Please add some detail about the selection and recruitment process for the participants and clarify the time interval between completion of the prior TPT to study participation as this could influence choices.

**RESPONSE:** We added the following detail in about the recruitment process under procedures (l.121-123):

**“**Consecutive potential participants were approached in the clinic waiting area by an HIV peer counsellor, with additional efforts to recruit persons who had never taken TPT and men. Participants received 20,000 Ugandan Shillings (about 5 USD).”

Unfortunately, we did not collect information on the timing of prior TPT, other than that it had been completed at least a year ago, as this was an eligibility criterion. However, about 30% of participants had taken 3HP, which first became available at the clinic through our implementation study “3HP Options Trial” in July 2020. Our DCE was performed as a sub-study and recruited participants from July to November 2022. Thus, 30% of participants had recently taken 3HP, within the last two years. We have added information relating to this in the discussion section (l.313-318):

“Thus, the high acceptability in our study may reflect … familiarity of *participants* with TPT *and specifically 3HP; about 30% of participants had previously taken 3HP, which was introduced at the clinic approximately two years before the present study.*”

26. Procedures

Please clarify whether participants received any standardized explanation of regimen attributes prior to completing the survey, and how understanding of the explanations was assessed.

**RESPONSE:** Thank you for the opportunity to clarify. We have specified the following in the manuscript (l.124-125):

**“**Before starting, participants were provided with general information on latent TB, and the DCE attributes and levels were explained with a structured flipbook, followed by a practice survey task.”

The flipbook is available as appendix B in the following preprint: [doi.org/10.1101/2023.09.13.23295043](https://doi.org/10.1101/2023.09.13.23295043) Although the preprint is now published in JIAS ([doi.org/10.1002/jia2.26390](https://doi.org/10.1002/jia2.26390)), this appendix was not published with the article, as we were requested to remove it. The flipbook had a participant and an interviewer-facing side.

Furthermore, we also specified that the survey and attributes were (l.128):

“iteratively refined through pilot testing with 29 patients [15].”

To assess understanding of the explanations, in this pilot, the participants were asked to explain attributes in their own words. The structured survey we used to collect information in the pilot is available as appendix A in the same preprint.

27. DCE design

Line 110: italicise the survey text to highlight that it is directly copied from the survey, and increase readability.

Line 118: As above, highlight survey text to increase readability. Secondly, please clarify there is text missing in the quoted segment, as I see use of an ellipsis.

**RESPONSE:** Thank you, we have italicized the quotes as suggested (now l.140 and l. 148). For clarity, we provided the entire quote instead of the abbreviated one (l. 148-151).

28. Statistical analysis

Please provide a brief explanation of hierarchical Bayesian estimation for readers less familiar with DCE methodology.

**RESPONSE:** Thank you for this suggestion. We have included the following brief explanation (l.164-166):

**“**These weights are estimated by assuming that (1) each participant has their own set of preference weights, and (2) these individual preferences are drawn from a broader distribution across the sample, which in turn informs each participant's estimates.”

29. Results

Line 229: Close bracket after “(Longer time on ART decreased the odds of refusing any regimen (OR=0.72 per 5 years, 95% CI 0.54 – 0.97”

**RESPONSE:** Thank you for catching this. In response to comments made by reviewer 1, we have removed this section.

Discussion:

30. A large majority of participants (over 90%) had previously received TPT. This may have influenced both familiarity with TPT and willingness to accept treatment. While the authors acknowledge this limitation, it would strengthen the paper to:

- Provide additional discussion on how prior exposure to TPT might bias preferences
- Clarify whether previous regimen experience (e.g., 6H vs 3HP) influenced responses
- Discuss implications for TPT-naive populations

**RESPONSE:** Thank you for pointing to this important limitation. Interestingly, we did not find meaningful differences in the predicted regimen preferences depending on previous experience of 6H vs 3HP, as shown in Tables S4 and S5. It is possible that this is due to measuring the preferences of attributes and levels in a neutral manner, rather than labeled, existing regimens, removing potential bias due to familiarity with regimens. We also did not find meaningful differences compared to TPT-naïve participants (Tables S5 and S6), however, this could also be an underpowered analysis, as only 9% of our sample were TPT-naïve.

In theory, prior TPT experience may bias persons towards TPT, if they had a positive experience, or may bias them against future TPT, if they had a negative experience, including significant side effects. There are two important additional considerations regarding how these participants may differ from one another. First, TPT-naïve participants were rare at the clinic (we had to oversample to reach 9%) and thus they may be unique in some way – they may be harder to reach, less educated about TB, or more resistant to preventive care in general. Second, participants who had experience with 3HP may be more likely to participate in studies, as they likely received 3HP as part of our implementation study “3HP Options Trial”. Despite these potential differences in participants, we did not observe meaningful differences in preferences. We have therefore added the following in the discussion section (l.331-334):

“Prior TPT experience could in principle influence preferences in either direction, and our largely TPT-experienced sample may limit generalizability to TPT-naïve populations; however, we observed no meaningful differences in predicted choices across participants with prior 6H, prior 3HP, or no prior TPT experience.”

31. The finding that side effects did not meaningfully influence acceptability is interesting but somewhat counterintuitive. The discussion could be expanded to explore possible explanations, such as:

- Difficulty interpreting probability-based risk descriptions
- Trust in healthcare providers
- Hypothetical framing compared to actual experiences with side effects

**RESPONSE:** Thank you for the suggestion to expand on this discussion. We had previously mentioned the effect of trust in healthcare providers and peers, as observed in a qualitative study performed within the same implementation study this DCE was part of (l.320-324):

**“**In contrast to our finding that the risk of adverse events did not impact acceptability, this study also identified perceived 3HP safety as a facilitator for accepting 3HP [34]. This may suggest that recommendations and shared positive experiences from peers and healthcare workers carry greater weight than being presented with varying risks of mild and moderate or severe side effects, which may be difficult for individuals to meaningfully interpret when deciding to start TPT.”

To address your comment, we have mentioned the difficulty of interpreting probability-based risk descriptions and the hypothetical framing more explicitly (l.325-329:

“*Similarly, we previously reported that our presentation of side effects did not meaningfully affect the preferences of most participants in our DCE [15], possibly reflecting difficulty of interpreting probability-based risk information, despite our efforts to communicate risk with icon arrays. Unlike in our hypothetical choice*, qualitative insights indicate that experiencing side effects can lead to TPT discontinuation, particularly when symptoms interfere with daily activities and income generation [34].”

32. The study was conducted at a single large urban HIV clinic in Kampala. The discussion should more clearly address how preferences might differ in:

- Rural settings
- Newly diagnosed PLHIV
- Individuals not yet engaged in HIV care

**RESPONSE:** Unfortunately, given that TPT has historically primarily been offered only to people with HIV in well-resourced clinics (which tend to be in urban centers) – not only in Uganda but also in the rest of Africa – empirical data on how preferences differ by urban/rural setting, and among individuals not yet engaged in HIV care in such settings do not currently exist. For TB care, in a study on health-seeking behavior in rural Uganda, patients sought care late or avoided care, and health-seeking behavior was negatively affected by poverty, drug stock-outs, and fear of HIV-testing ([doi.org/10.5588/ijtld.10.0211](https://doi.org/10.5588/ijtld.10.0211)). We are also unaware of any studies that explicitly compare the preferences of newly diagnosed PLHIV versus those who are treatment-experienced (though TPT is commonly provided in this context). As such, since there is limited to no peer-reviewed evidence on which to base any such comparison, we have added to the Discussion (l.379-382):

**“***Third, our results may not be generalizable to rural areas, newly diagnosed PLHIV, and PLHIV who are not actively engaged in HIV care. Additional research to explore how preferences may vary across such populations would be valuable as access to TPT is expanded in the future.****”***

33. The findings support the rollout of 3HP fixed-dose combinations. However, the discussion could expand on the practical implications for TB and HIV programs in Uganda and broadly, such as:

- Counselling approaches when multiple regimens are available
- Implementation of patient choice models in routine care

Linking the findings more explicitly to programmatic decision-making would strengthen the policy relevance.

**RESPONSE:** Thank you for pointing this out. We have added the following text to the discussion section (l.363-367):

**“**Offering regimen choices may come with challenges for TB and HIV programs regarding implementation, as more counseling and tailored support to complete each regimen may be needed. For example, in the 3HP Options Trial, a decision aid was used to support participants in choosing between directly observed and self-administered therapy to complete 3HP [34,39].”

**Reviewer #3:**

34. This is a very well written manuscript that addresses an important research question. I have no substantial comments, except that I would have liked the words "Shares of Preference Model using the Sawtooth Choice Simulator tool" to be explained briefly. I realise that there is a citation, and with some effort the curious reader could research what this tool does. However, I do think it would be preferable to the reader to have a few sentences here that explains briefly what this tool does.

**RESPONSE:** Thank you for your positive review. We have included the following brief explanation about the Shares of Preference Model (l.168-171):

**“**The Shares of Preference Model computes the utility for each regimen (adding up participants’ individual part-worth utilities), converts the utilities into choice probabilities, and aggregates the probabilities across all participants to predict which proportion of the study population would choose which regimen.”
