# Supplementary material for: Predicted choice and acceptability of regimens for tuberculosis preventive treatment among people living with HIV in Uganda – A discrete choice experiment
Source: PLOS Glob Public Health. 2026 Sep 10;6(9):e0005630. doi: 10.1371/journal.pgph.0005630 (PMC13561322; doi:10.1371/journal.pgph.0005630)
Supplement: Peer review history

# Peer review: Decision Letter 0

**DOI:** 10.1371/journal.pgph.0005630.r001

**Authors:** Graeme Hoddinott

22 Mar 2026

PGPH-D-25-03555

Predicted choice and acceptability of regimens for tuberculosis preventive treatment among people living with HIV in Uganda – a discrete choice experiment

PLOS Global Public Health

Dear Dr. Aschmann,

Thank you for submitting your manuscript to PLOS Global Public Health. After careful consideration, we feel that it has merit but does not fully meet PLOS Global Public Health’s publication criteria as it currently stands. Therefore, we invite you to submit a revised version of the manuscript that addresses the points raised during the review process.

==============================

EDITOR:

The reviewers have raised (in my view) relevant points of refinement. Some of these are methodological. I've classed this as a 'major' review because substantive changes may be required to the presentation of the manuscript. However, these all seem addressable to me and I would hope that you are encouraged to engage the reviewer comments in the spirit of refinement toward the best possible version of the manuscript.

==============================

Please submit your revised manuscript by 11 April 2026. If you will need more time than this to complete your revisions, please reply to this message or contact the journal office at globalpubhealth@plos.org. When you're ready to submit your revision, log on to https://www.editorialmanager.com/pgph/ and select the 'Submissions Needing Revision' folder to locate your manuscript file.

Please include the following items when submitting your revised manuscript:

- A letter that responds to each point raised by the editor and reviewer(s). You should upload this letter as a separate file labeled 'Response to Reviewers'.
- A marked-up copy of your manuscript that highlights changes made to the original version. You should upload this as a separate file labeled 'Revised Manuscript with Track Changes'.
- An unmarked version of your revised paper without tracked changes. You should upload this as a separate file labeled 'Manuscript'.

Guidelines for resubmitting your figure files are available below the reviewer comments at the end of this letter.

We look forward to receiving your revised manuscript.

Kind regards,

Graeme Hoddinott, Ph.D

Academic Editor

PLOS Global Public Health

**Journal Requirements:**

1. Please include a complete copy of PLOS’ questionnaire on inclusivity in global research in your revised manuscript. Our policy for research in this area aims to improve transparency in the reporting of research performed outside of researchers’ own country or community. The policy applies to researchers who have travelled to a different country to conduct research, research with Indigenous populations or their lands, and research on cultural artefacts. The questionnaire can also be requested at the journal’s discretion for any other submissions, even if these conditions are not met. Please find more information on the policy and a link to download a blank copy of the questionnaire here: https://journals.plos.org/globalpublichealth/s/best-practices-in-research-reporting. Please upload a completed version of your questionnaire as Supporting Information when you resubmit your manuscript.

2. Please amend your detailed Financial Disclosure statement. This is published with the article. It must therefore be completed in full sentences and contain the exact wording you wish to be published.

i. Please clarify all sources of financial support for your study. List the grants, grant numbers, and organizations that funded your study, including funding received from your institution. Please note that suppliers of material support, including research materials, should be recognized in the Acknowledgements section rather than in the Financial Disclosure.

ii. State the initials, alongside each funding source, of each author to receive each grant. For example: "This work was supported by the National Institutes of Health (####### to AM; ###### to CJ) and the National Science Foundation (###### to AM)."

iii. State what role the funders took in the study. If the funders had no role in your study, please state: “The funders had no role in study design, data collection and analysis, decision to publish, or preparation of the manuscript.”

iv. If any authors received a salary from any of your funders, please state which authors and which funders.

3. In the online submission form, you indicated that “Deidentified individual participant data that support the findings of this study are available from the corresponding author upon reasonable request after approval of a proposal.”.

All PLOS journals now require all data underlying the findings described in their manuscript to be freely available to other researchers, either

1. In a public repository,

2. Within the manuscript itself, or

3. Uploaded as supplementary information.

This policy applies to all data except where public deposition would breach compliance with the protocol approved by your research ethics board. If your data cannot be made publicly available for ethical or legal reasons (e.g., public availability would compromise patient privacy), please explain your reasons by return email and your exemption request will be escalated to the editor for approval. Your exemption request will be handled independently and will not hold up the peer review process, but will need to be resolved should your manuscript be accepted for publication. One of the Editorial team will then be in touch if there are any issues.

4. Some material included in your submission may be copyrighted. According to PLOS’s copyright policy, authors who use figures or other material (e.g., graphics, clipart, maps) from another author or copyright holder must demonstrate or obtain permission to publish this material under the Creative Commons Attribution 4.0 International (CC BY 4.0) License used by PLOS journals. Please closely review the details of PLOS’s copyright requirements here: PLOS Licenses and Copyright. If you need to request permissions from a copyright holder, you may use PLOS's Copyright Content Permission form.

Please respond directly to this email or email the journal office and provide any known details concerning your material's license terms and permissions required for reuse, even if you have not yet obtained copyright permissions or are unsure of your material's copyright compatibility.

Potential Copyright Issues:

a. Figure S1: Please confirm whether you drew the images / clip-art within the figure panels by hand. If you did not draw the images, please provide (a) a link to the source of the images or icons and their license / terms of use; or (b) written permission from the copyright holder to publish the images or icons under our CC-BY 4.0 license. Alternatively, you may replace the images with open source alternatives. See these open source resources you may use to replace images / clip-art:

- https://commons.wikimedia.org

- https://openclipart.org/

If the reviewer comments include a recommendation to cite specific previously published works, please review and evaluate these publications to determine whether they are relevant and should be cited. There is no requirement to cite these works unless the editor has indicated otherwise.

**Additional Editor Comments (if provided):**

[Note: HTML markup is below. Please do not edit.]

Reviewers' comments:

Reviewer's Responses to Questions

**Comments to the Author**

1. Does this manuscript meet PLOS Global Public Health’s publication criteria?

Reviewer #1: Partly

Reviewer #2: Yes

Reviewer #3: Yes

**********

2. Has the statistical analysis been performed appropriately and rigorously?

Reviewer #1: No

Reviewer #2: Yes

Reviewer #3: I don't know

**********

3. Have the authors made all data underlying the findings in their manuscript fully available (please refer to the Data Availability Statement at the start of the manuscript PDF file)?

The PLOS Data policy

Reviewer #1: No

Reviewer #2: Yes

Reviewer #3: Yes

**********

4. Is the manuscript presented in an intelligible fashion and written in standard English?

Reviewer #1: Yes

Reviewer #2: Yes

Reviewer #3: Yes

**********

**Reviewer #1:**  Dear Authors,

This manuscript and the findings from you study are important additions to the literature. However, I believe that the manuscript needs to be strengthened, and the approach to the analysis reconsidered before the paper can be published. I have provided detailed feedback to help strengthen the manuscript and refine the approach to the data analysis for your consideration, attached. I hope that this review will be useful for strengthening the manuscript and the implications of your work for policy, guidelines and practice.

**Reviewer #2:**  Overall: Overall, this is a well-conducted and policy-relevant study that contributes important insights into patient preferences for TB preventive treatment among PLHIV. The results strongly support the scale-up of shorter, simpler regimens, particularly 3HP fixed-dose combinations, to improve uptake and person-centred care in high-burden settings. The discrete choice experiment is well-designed and allows quantification of patient preferences. The study aligns with growing emphasis on person-centred TB prevention. However, several areas would benefit from clarification and further discussion, particularly regarding generalizability, interpretation of acceptability results, and implications for programmatic implementation.

Abstract:

1. The first sentence can be improved for better flow, as it currently sounds incomplete. The sentence can be written as “Little is known about people living with HIV’s preferences for different TPT regimens, or the conditions under which they’d accept treatment.”

Setting and participants

1. Please add some detail about the selection and recruitment process for the participants and clarify the time interval between completion of the prior TPT to study participation as this could influence choices.

Procedures

1. Please clarify whether participants received any standardized explanation of regimen attributes prior to completing the survey, and how understanding of the explanations was assessed.

DCE design

1. Line 110: italicise the survey text to highlight that it is directly copied from the survey, and increase readability.

2. Line 118: As above, highlight survey text to increase readability. Secondly, please clarify there is text missing in the quoted segment, as I see use of an ellipsis.

Statistical analysis

1. Please provide a brief explanation of hierarchical Bayesian estimation for readers less familiar with DCE methodology.

Results

1. Line 229: Close bracket after “(Longer time on ART decreased the odds of refusing any regimen (OR=0.72 per 5 years, 95% CI 0.54 – 0.97”

Discussion:

1. A large majority of participants (over 90%) had previously received TPT. This may have influenced both familiarity with TPT and willingness to accept treatment. While the authors acknowledge this limitation, it would strengthen the paper to:

• Provide additional discussion on how prior exposure to TPT might bias preferences

• Clarify whether previous regimen experience (e.g., 6H vs 3HP) influenced responses

• Discuss implications for TPT-naive populations

2. The finding that side effects did not meaningfully influence acceptability is interesting but somewhat counterintuitive. The discussion could be expanded to explore possible explanations, such as:

• Difficulty interpreting probability-based risk descriptions

• Trust in healthcare providers

• Hypothetical framing compared to actual experiences with side effects

3. The study was conducted at a single large urban HIV clinic in Kampala. The discussion should more clearly address how preferences might differ in:

• Rural settings

• Newly diagnosed PLHIV

• Individuals not yet engaged in HIV care

4. The findings support the rollout of 3HP fixed-dose combinations. However, the discussion could expand on the practical implications for TB and HIV programs in Uganda and broadly, such as:

• Counselling approaches when multiple regimens are available

• Implementation of patient choice models in routine care

Linking the findings more explicitly to programmatic decision-making would strengthen the policy relevance.

**Reviewer #3:**  This is a very well written manuscript that addresses an important research question. I have no substantial comments, except that I would have liked the words "Shares of Preference Model using the Sawtooth Choice Simulator tool" to be explained briefly. I realise that there is a citation, and with some effort the curious reader could research what this tool does. However, I do think it would be preferable to the reader to have a few sentences here that explains briefly what this tool does.

**********

what does this mean?). If published, this will include your full peer review and any attached files.

**Do you want your identity to be public for this peer review?**  If you choose “no”, your identity will remain anonymous but your review may still be made public.

For information about this choice, including consent withdrawal, please see our Privacy Policy

Reviewer #1: No

Reviewer #2: No

Reviewer #3: No

**********

[NOTE: If reviewer comments were submitted as an attachment file, they will be attached to this email and accessible via the submission site. Please log into your account, locate the manuscript record, and check for the action link "View Attachments". If this link does not appear, there are no attachment files.]

Attachment Submitted filename: Review comments_PGPH-D-23-03555.docx

Attachment Submitted filename: PGPH_Predicted choice and acceptability of regimens for TPT.docx

# Author comment: Author response to Decision Letter 1

**DOI:** 10.1371/journal.pgph.0005630.r002

29 Jun 2026

Attachment Submitted filename: R1_ResponseLetter_DCE_acceptability_TPT_20260623.docx

# Peer review: Decision Letter 1

**DOI:** 10.1371/journal.pgph.0005630.r003

**Authors:** Graeme Hoddinott

3 Aug 2026

Predicted choice and acceptability of regimens for tuberculosis preventive treatment among people living with HIV in Uganda – a discrete choice experiment

PGPH-D-25-03555R1

Dear Dr. Aschmann,

We are pleased to inform you that your manuscript 'Predicted choice and acceptability of regimens for tuberculosis preventive treatment among people living with HIV in Uganda – a discrete choice experiment' has been provisionally accepted for publication in PLOS Global Public Health.

Before your manuscript can be formally accepted you will need to complete some formatting changes, which you will receive in a follow up email. A member of our team will be in touch with a set of requests.

Please note that your manuscript will not be scheduled for publication until you have made the required changes, so a swift response is appreciated.

IMPORTANT: The editorial review process is now complete. PLOS will only permit corrections to spelling, formatting or significant scientific errors from this point onwards. Requests for major changes, or any which affect the scientific understanding of your work, will cause delays to the publication date of your manuscript.

If your institution or institutions have a press office, please notify them about your upcoming paper to help maximize its impact. If they'll be preparing press materials, please inform our press team as soon as possible -- no later than 48 hours after receiving the formal acceptance. Your manuscript will remain under strict press embargo until 2 pm Eastern Time on the date of publication. For more information, please contact globalpubhealth@plos.org.

Thank you again for supporting Open Access publishing; we are looking forward to publishing your work in PLOS Global Public Health.

Best regards,

Graeme Hoddinott, Ph.D

Academic Editor

PLOS Global Public Health

***********************************************************

Reviewer Comments (if any, and for reference):

Reviewer's Responses to Questions

**Comments to the Author**

Reviewer #1: All comments have been addressed

Reviewer #3: All comments have been addressed

**********

publication criteria?

Reviewer #1: Yes

Reviewer #3: Yes

**********

3. Has the statistical analysis been performed appropriately and rigorously?

Reviewer #1: Yes

Reviewer #3: Yes

**********

4. Have the authors made all data underlying the findings in their manuscript fully available (please refer to the Data Availability Statement at the start of the manuscript PDF file)?

The PLOS Data policy

Reviewer #1: No

Reviewer #3: Yes

**********

5. Is the manuscript presented in an intelligible fashion and written in standard English?

Reviewer #1: Yes

Reviewer #3: Yes

**********

Reviewer #1: (No Response)

Reviewer #3: (No Response)

**********

what does this mean?). If published, this will include your full peer review and any attached files.

**Do you want your identity to be public for this peer review?**  If you choose “no”, your identity will remain anonymous but your review may still be made public.

For information about this choice, including consent withdrawal, please see our Privacy Policy

Reviewer #1: No

Reviewer #3: No

**********
